# A Van Der Waals Broadband Infrared Optical Synapse Enabling Orientation Detection

**DOI:** 10.1002/advs.202507530

**Published:** 2025-09-30

**Authors:** Dan Guo, Wenjing Li, Pingfan Gu, Weikang Dong, Xuyan Rui, Kenji Watanabe, Takashi Taniguchi, Yu Ye, Fawei Zheng, Jiadong Zhou, Shoujun Zheng

**Affiliations:** ^1^ Centre for Quantum Physics Key Laboratory of Advanced Optoelectronic Quantum Architecture and Measurement (MOE) School of Physics Beijing Institute of Technology Beijing 100081 China; ^2^ State Key Laboratory for Artificial Microstructure & Mesoscopic Physics and Frontiers Science Center for Nano‐Optoelectronics School of Physics Peking University Beijing 100871 China; ^3^ National Institute for Materials Science 1‐1 Namiki Tsukuba 303‐0044 Japan; ^4^ Faculty of Marine Science and Technology Beijing Institute of Technology Zhuhai Guangdong 519088 China

**Keywords:** 2D materials, infrared optical synapse, interlayer coupling, orientation detection, van der Waals heterostructure

## Abstract

A vision system with efficient infrared‐sensitive optical synapses is crucial for enabling infrared radiation detection and target recognition for some predators hunting in the dark. Current 2D synaptic devices typically adopt the strategy of charge trap and release to achieve intelligent sensing and visual recognition. However, the response wavelength is limited in a narrow range (typically in visible) due to the intrinsic bandgap of these 2D materials. In this work, a broadband infrared optoelectronic synaptic device based on a few‐layer graphene/CrOCl/few‐layer graphene van der Waals (vdW) heterostructure is reported, featuring tunable spike timing‐dependent plasticity and a broadband response range from the visible to the infrared (520–2000 nm). The broadband synaptic response in the tunneling device is attributed to the modulation of the tunneling barrier by strong interfacial coupling and charge transfer‐induced long‐wavelength charge order at the vdW interface. Integrated with reservoir computing technique, the tunneling device can efficiently detect images in different orientations, achieving a recognition accuracy exceeding 98%, and judge the possible escape directions of a mouse. This work not only allows us to explore broadband optical synapses by controlling the vdW interfacial coupling but also offers a promising solution for developing advanced infrared detection systems.

## Graphical abstract

An infrared optoelectronic synapse based on a graphene/CrOCl/graphene van der Waals heterostructure is reported to possess a broadband synaptic response range from the visible to the infrared (520–2000 nm), which can be used to achieve orientation and motion detections of a target mimicing the pit organ of a pit vapor.

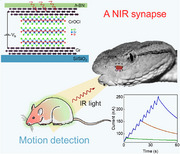

## 1 Introduction

Brain‐inspired neuromorphic computing is increasingly being explored as an alternative for efficient information processing and transmission^[^ ^1^, ^2^ ^]^ to address the bottleneck issues of low efficiency and high energy consumption caused by the separation of memory and computational units in von Neumann architectures.^[^ ^3^ ^]^ Synapses are the fundamental units of the brain's neural system responsible for detection, learning, and computation, playing a critical role in information transfer and processing.^[^ ^4^ ^]^ Artificial synapses are desired for the on‐chip circuit to mimic the plasticity of synapses for detecting and learning environmental signals, such as excitatory postsynaptic current (EPSC).^[^ ^5^, ^6^ ^]^ In recent years, electrical and optical artificial synaptic devices based on 2D materials have been widely reported for neuromorphic computing.^[^ ^7^, ^8^ ^]^ However, these devices majorly rely on charge trap and release processes originating from defects or floating gates, leading to device instability, functional degradation, and complex modulation.^[^ ^9^ ^]^ Therefore, it is essential to develop 2D devices with advanced mechanisms to simulate synaptic behaviors effectively.

2D optical synaptic devices with visual recognition are expected to be applied in many practical fields, such as the Internet of Things smart sensors, biomedical electronics, and robotics.^[^ ^10^ ^]^ To date, numerous 2D optical synaptic devices have been reported to be capable of visual recognition based on various operational mechanisms, including persistent photoconductive effects,^[^ ^11^, ^12^ ^]^ photo‐gating effects,^[^ ^13^ ^]^ material defects and charge‐trapping effects,^[^ ^14^ ^]^ photogenerated carrier generation and recombination,^[^ ^15^ ^]^ ferroelectric‐induced separation of photogenerated carriers,^[^ ^16^ ^]^ and tunneling of photogenerated carriers.^[^ ^17^ ^]^ However, most of these devices exhibit optical responses in the ultraviolet (UV) and visible (VIS) ranges due to the intrinsic band gap of 2D materials. Research on the optical synapse operating in the infrared (IR) range remains limited.

The key to designing IR optical synapse devices is to effectively modulate the lifetime of carriers generated by the IR photons. First, the 2D materials must exhibit a high photoresponse in the IR range. Although black phosphorus (BP) possesses a narrow band gap suitable for IR detection, its applications are severely limited by its susceptibility to oxidation.^[^ ^18^ ^]^ Graphene is another candidate for IR detection due to its gapless band structure;^[^ ^19^, ^20^ ^]^ however, the rapid recombination of hot charge carriers restricts its use in optoelectronic synapses.^[^ ^21^, ^22^ ^]^ Second, van der Waals (vdW) heterostructures provide a promising platform for modulating photon‐generated carriers and enabling synaptic functions driven by interlayer charge transport and strong interfacial coupling effect. For example, as an indirect bandgap material, CrOCl typically has a large bandgap (≈2.77 eV),^[^ ^23^ ^]^ which is consistent with our absorbance spectrum result of 2.3 eV in Figure S1 (Supporting Information). The wide bandgap makes it a good insulator, which also means it is not suitable for direct detection of infrared light. However, vdW heterostructures combined CrOCl with other 2D materials, such as graphene and transition metal dichalcogenides (TMDs), can be used to modulate their electronic properties via strong interlayer coupling and potentially extend to infrared light detection. Specifically, vdW interactions between CrOCl and other 2D materials have been reported to reconfigure the polarity of 2D TMDs^[^ ^24^ ^]^ and achieve large on/off ratios in graphene field effect transistors.^[^ ^25^, ^26^ ^]^ The strong interlayer coupling and charge transfer between CrOCl and 2D material allow us to modulate optoelectronic properties at the vdW interface. Therefore, vdW heterostructures can be employed to develop air‐stable IR optical synapse devices, which can meet the demands of various practical applications, such as intelligent imaging, autonomous driving, and space communications.

In this study, we fabricated a few‐layer graphene/CrOCl/few‐layer graphene (FLG/CrOCl/FLG) vdW heterostructure to achieve broadband IR optical synapse. Compared to previously reported synaptic devices, our device demonstrates efficient simulation of optical synaptic behaviors with tunable spike‐timing‐dependent plasticity (STDP), spike‐number‐dependent plasticity (SNDP), spike‐rate‐dependent plasticity (SRDP), and paired pulse facilitation (PPF) over a broad spectral range from the VIS to the IR (520–2000 nm). Theoretical calculations reveal that the strong interfacial coupling at the graphene/CrOCl interface induces the formation of long‐wavelength charge order and reduces the tunneling barrier under illumination. Integrated with reservoir computing (RC), the optical synapse is capable of effectively sensing IR signals in different orientations, achieving an accuracy exceeding 98%. Our IR synaptic device demonstrates that the vdW heterostructure is a promising platform for designing high‐performance optical neuromorphic devices and exploring potential applications in IR intelligent imaging and retinomorphic computing.

## 2 Results and Discussion

### 2.1 Biomimetic IR Perception of Snake

**Figure 1:**
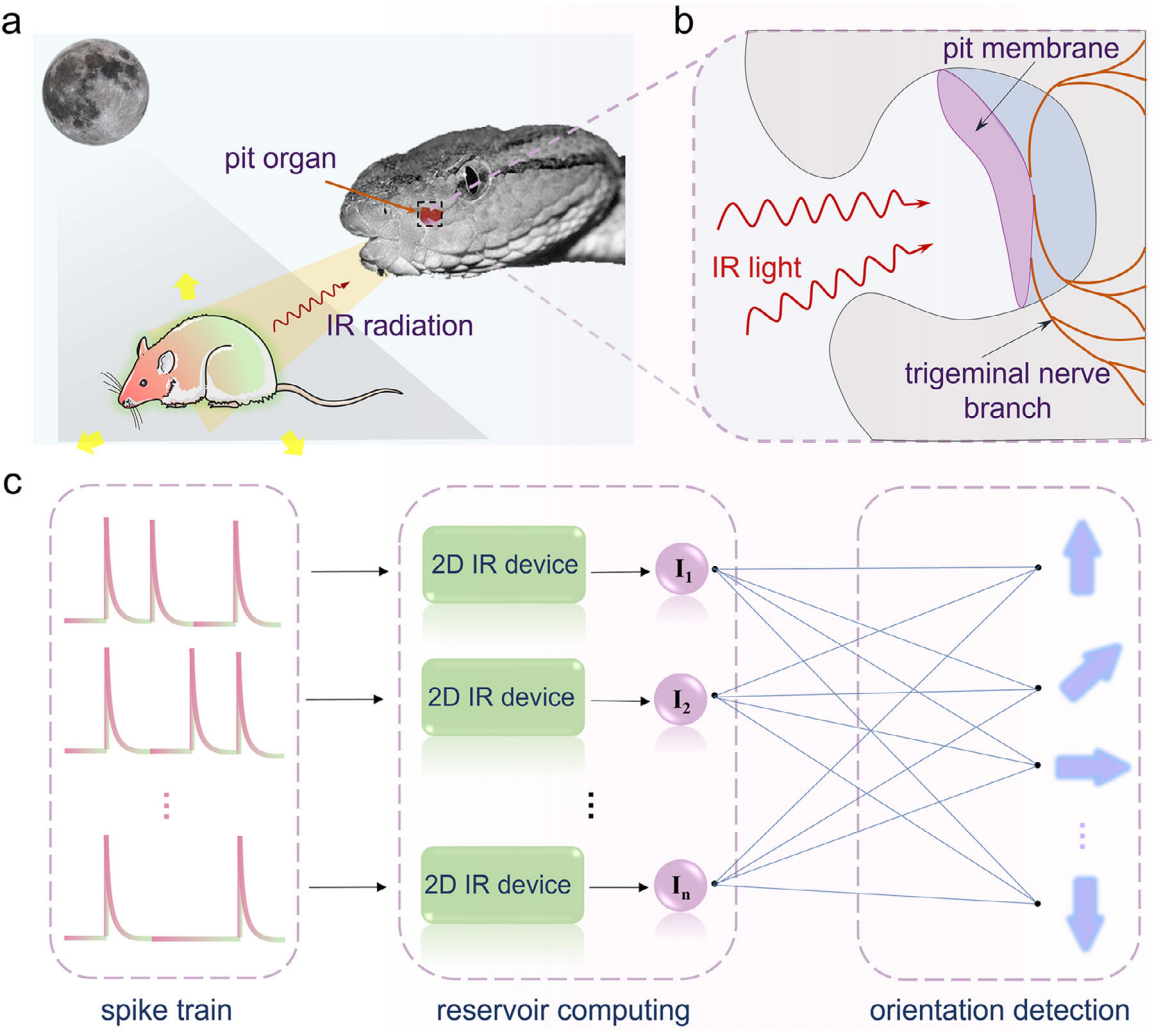
Detection of a prey through an infrared optical synapse. a) The infrared radiation perception of a pit viper utilizing the pit organ located between the mouth and eyes to detect the prey movement direction in the darkness. b) The detailed structures of a pit organ composed of the pit membrane (a thin membrane‐like tissue with the ability to sense infrared radiation) and the trigeminal nerve branch (transmits the infrared radiation signals detected by the pit organ to the brain, enabling the snake to perceive the location and movement direction of warm‐blooded prey). c) Recognition processing of infrared radiation pulse trains using reservoir computing for the image classification.

An IR‐sensitive vision system is essential for IR radiation detection and target imaging in some nocturnal animals such as pit vapor and vampire bat (shown in **Figure** **1**a). These predators possess a thermosensitive pit organ (consisting of a pit membrane and trigeminal nerves, shown in Figure 1b) that enables them to detect IR radiation and perceive temperature variations even in dark environments.^[^ ^27^, ^28^ ^]^ The trigeminal nerve plays a crucial role in detecting, transmitting, and processing IR signals through the famous transient receptor potential cation channel A1 (TRPA1) channel.^[^ ^29^, ^30^ ^]^ Based on this, an IR optical synaptic device can theoretically replicate the function of the pit organ by responding to and processing IR light pulses to capture IR targets. Integrated with RC, the IR optical synapse can theoretically detect orientations in response to synaptic stimulation, like tracking the directions of prey movement (Figure 1c).

**Figure 2:**
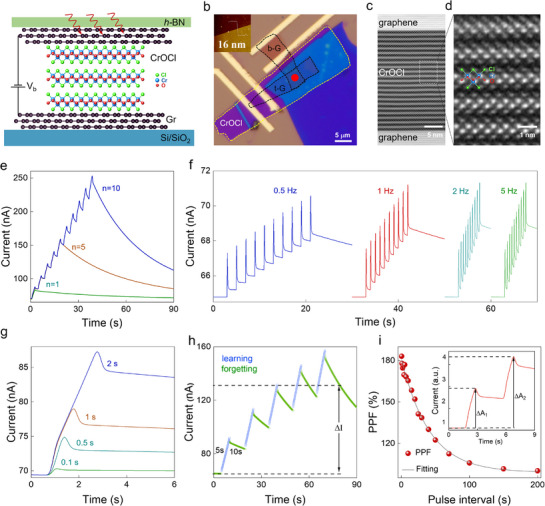
The structure of the device and synaptic plasticity under 1550 nm laser optical stimulation. a) Schematic diagram of the few‐layer graphene/CrOCl/few‐layer graphene optoelectronic device, which is fully encapsulated in hBN. b) Optical image of the device. few‐layer graphene and CrOCl are highlighted with black and yellow dashed lines. Inset shows the AFM morphology of the CrOCl, with a thickness of 16 nm. c) Cross‐sectional TEM image of the device. Scale bar is 5 nm. d) Enlarged cross‐sectional STEM‐HAADF image in c showing the lattice structure of CrOCl. Scale bar is 1 nm. e) SNDP triggered by pulse numbers where a single optical pulse is 2 s and the power is 51 µW at V_g_ = 0 V and V_b_ = 1 V. f) SRDP triggered by different frequencies of optical pulse from 0.5 to 5 Hz s with the power of 51 µW at V_g_ = 0 V and V_b_ = 1 V. g) Pulse duration‐dependent photoresponse with the power of 51 µW at V_g_ = 0 V and V_b_ = 1 V. h) Memory characteristics induced by optical pulses for simulating human learning‐forgetting‐relearning behavior. i) Dependence of the PPF ratio (defined as ΔA_2_/ΔA_1_ × 100%) on the pulse interval with the light power of 51 µW and pulse duration of 1 s at V_g_ = 0 V and V_b_ = 1 V. Inset shows the PPF behavior stimulated by a pair of optical pulses.

To detect IR signals, we fabricated a FLG/CrOCl/FLG tunneling device and measured the tunneling current under IR light illumination, as illustrated in **Figure** **2**a, in which few‐layer graphene is commonly employed in IR detectors due to its gapless band structure and high response to IR light. CrOCl, an emerging antiferromagnetic insulator that has been applied in heat dissipation devices^[^ ^31^ ^]^ and memory devices^[^ ^32^ ^]^ due to its low‐symmetry crystal structure, large magnetoelastic coupling effects, and vacancy‐tunable bandgap,^[^ ^33^ ^]^ is used as both a tunneling barrier and an interfacial coupling layer with graphene. The strong interfacial coupling between graphene and CrOCl has been exploited to tune the quantum Hall state and open a gap in graphene.^[^ ^25^ ^]^

In our FLG/CrOCl/FLG tunneling device (optical image shown in Figure 2b), a bias voltage is applied to the bottom graphene layer, and the tunneling current is measured under various light pulse stimulations. The device's active region is encapsulated by a ≈30 nm thick hexagonal boron nitride (*h*‐BN) (highlighted by the red circle in Figure 2b). Figure 2c presents the transmission electron microscopy (TEM) image of the cross‐section of the vdW heterostructure, where both graphene and CrOCl can be clearly distinguished. CrOCl is identified as 22 layers thick, which is consistent with our atomic force microscopy (AFM) measurements (the inset in Figure 2b shows the CrOCl thickness of 16 nm). The crystal structure of CrOCl is shown in the scanning transmission electron microscopy high‐angle annular dark‐field (STEM‐HAADF) image in Figure 2d, revealing its high crystalline quality.

We performed *I–V* test on the vdW tunneling device in the dark without applying gate voltage, which shows a ≈80 nA tunneling current at a ±1 V bias (Figure S2, Supporting Information). For comparison, we fabricated two Au/CrOCl/Au devices with CrOCl thicknesses of 45 and 6.5 nm, respectively (Figure S3, Supporting Information). Both devices exhibited insulating behavior at a ±1 V bias under different gate voltages, demonstrating that CrOCl is a good insulator, consistent with reported literatures.^[^ ^34^ ^]^ The difference in conductance between the two types of tunneling devices indicates a strong interfacial coupling effect and efficient charge transfer at the vdW interface of graphene and CrOCl, which significantly modifies the tunneling barrier.

### 2.2 IR Synaptic Plasticity of the Tunneling Device

To demonstrate the opto‐synaptic plasticity of the vdW heterostructure, we applied 1550 nm laser pulses with a 2 s pulse width to the tunneling device (shown in Figure 2e). Unless otherwise specified, all measurements in Figure 2 were conducted under these conditions, with 1550 nm light illumination at a bias of 1 V. In biological vision systems, SNDP is the essential function for processing time‐dependent signals by modulating both the sign and magnitude of synaptic strength, and SNDP was achieved by applying successive pulse stimuli in our device (Figure 2e). As the number of light pulses increases, the synaptic weight (A_n_/A_1_) continuously increases, and the ratio of A_60_/A_1_ approaches 16 (Figure S4, Supporting Information), suggesting the potential for improved contrast in neuromorphic imaging and preprocessing applications. Figure 2f illustrates the SRDP performance of the device, transitioning from 0.5 to 5 Hz under ten light pulses at varying frequencies, and the peak value of EPSC increases with the frequency of the laser pulses. Moreover, the EPSC increase at a specific frequency is evaluated in terms of gain, which is defined as the ratio of the maximum PSC induced by the tenth light pulse (A_10_) to the maximum current induced by the first light pulse (A_1_), and we observe that the gain (A_10_/A_1_) increases with the frequency of the pulses Figure S5, Supporting Information).

Unlike typical photodetectors, the photocurrent in our device continuously increases with the light illumination power and gradually recovers slowly once the light stimulus is removed. The magnitude of the photocurrent increases approximately linearly with light intensity (Figure S6, Supporting Information), and the device exhibits a longer rise time and falling time, which is attributed to the continuous modulation of the tunneling barrier by photogenerated carriers produced within the top graphene layer. Furthermore, the photocurrent increases linearly with pulse duration (Figure 2g; Figure S7, Supporting Information) and demonstrates clear STDP with a tunable EPSC. Five cycles of on/off light pulses with 5 s illumination time and 10 s shut‐off time were used to test the learning and forgetting processes (Figure 2h). The current increases from 65 to 92 µA during the first learning phase and then decreases to a value slightly higher than the initial state. The subsequent re‐learning process further strengthens the memory function, and the ∆I difference between the first and fifth decayed currents represents the repeated learning process, which follows the Ebbinghaus forgetting curve^[^ ^35^, ^36^ ^]^ of the human brain about the learning and forgetting behaviors.

The paired pulse facilitation (PPF) index, defined as ΔA_2_/ΔA_1_ × 100%, is commonly used to quantify short‐term plasticity responses between neurons, particularly the changes in synaptic response under consecutive stimuli. The PPF triggered by the 1550 nm light illumination in our device is 184% (shown in Figure 2i), indicating that the response to the second pulse is 84% greater than that of the first. This reflects that our device exhibits an efficient STDP, which is critical for information processing as well as the short‐time formation of learning and memory in the nervous system. These results suggest that our device serves as an ideal IR optical synapse with tunable EPSC.

### 2.3 Broadband Synaptic Response from VIS to IR

**Figure 3:**
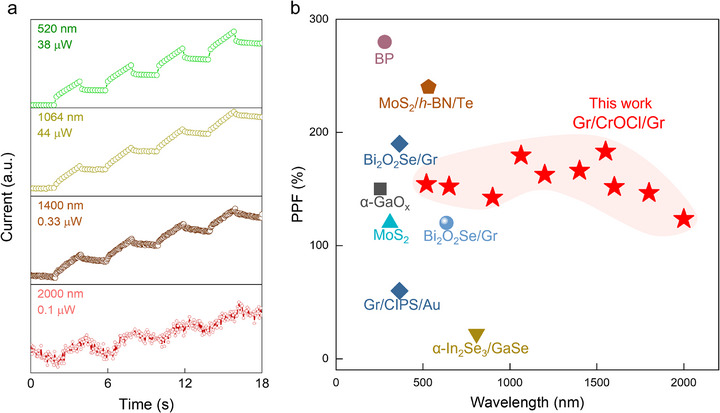
Broadband synaptic response of the graphene/CrOCl/graphene device. a) Optical synaptic behavior of the device with light excitation of four wavelengths of 520, 1064, 1400, and 2000 nm at V_g_ = 0 V and V_b_ = 1 V. b) Summary of the PPF of previously reported optical synaptic devices^[^ ^8^, ^14^, ^41^, ^48^, ^49^, ^50^ ^]^ and our device. Our device exhibits significant synaptic plasticity in the infrared range.

Artificial synapses, in conjunction with visual recognition, have become an inevitable development trend for future technologies, particularly in areas such as night vision imaging and autonomous driving. Most reported optical artificial synapses based on 2D materials operate within a narrow VIS spectrum.^[^ ^12^, ^37^, ^38^ ^]^ However, our device exhibits synaptic plasticity across a broad spectral range, extending from the VIS to the IR range. We tested our device under laser illumination at various wavelengths and observed significant optical synaptic plasticity at wavelengths of 520, 1064, 1400, and 2000 nm shown in **Figure** **3**a. The dependences of the EPSC on laser pulse number, frequency, power, and duration are consistent with those observed at 1550 nm (Figures S8–S11, Supporting Information). Additionally, the PPF also shows great tunability to pulse interval (Figure S12, Supporting Information). Figure 3b summarizes optical synaptic devices based on 2D materials (more reported 2D optical synapses are summarized in Table S1, Supporting Information). The enhanced synaptic characteristics of our device position it as a promising candidate for advancing nighttime imaging and recognition applications.

To investigate CrOCl thickness‐dependent synaptic responses, we fabricated tunneling devices with thicknesses of 2.5 and 40 nm (Figure S14, Supporting Information) and tested them with 520 and 1064 nm lasers. Similar to the 16 nm thickness, both the 2.5 and 40 nm thick tunneling devices demonstrated strong optical synaptic plasticity. We compared the test results of devices with three different CrOCl thicknesses with 520 and 1064 nm lasers, using identical test conditions (Table S2, Supporting Information). The dark currents of the tunneling devices are 900 µA at 2.5 nm (thin), 80 nA at 16 nm (medium), and 6.5 nA at 40 nm (thick), respectively. Devices with all three thicknesses demonstrated significant optical synaptic plasticity and only slight variations of ΔA_10_/ΔA_1_ and PPF.

### 2.4 Mechanisms for the Broadband Synaptic Response

**Figure 4:**
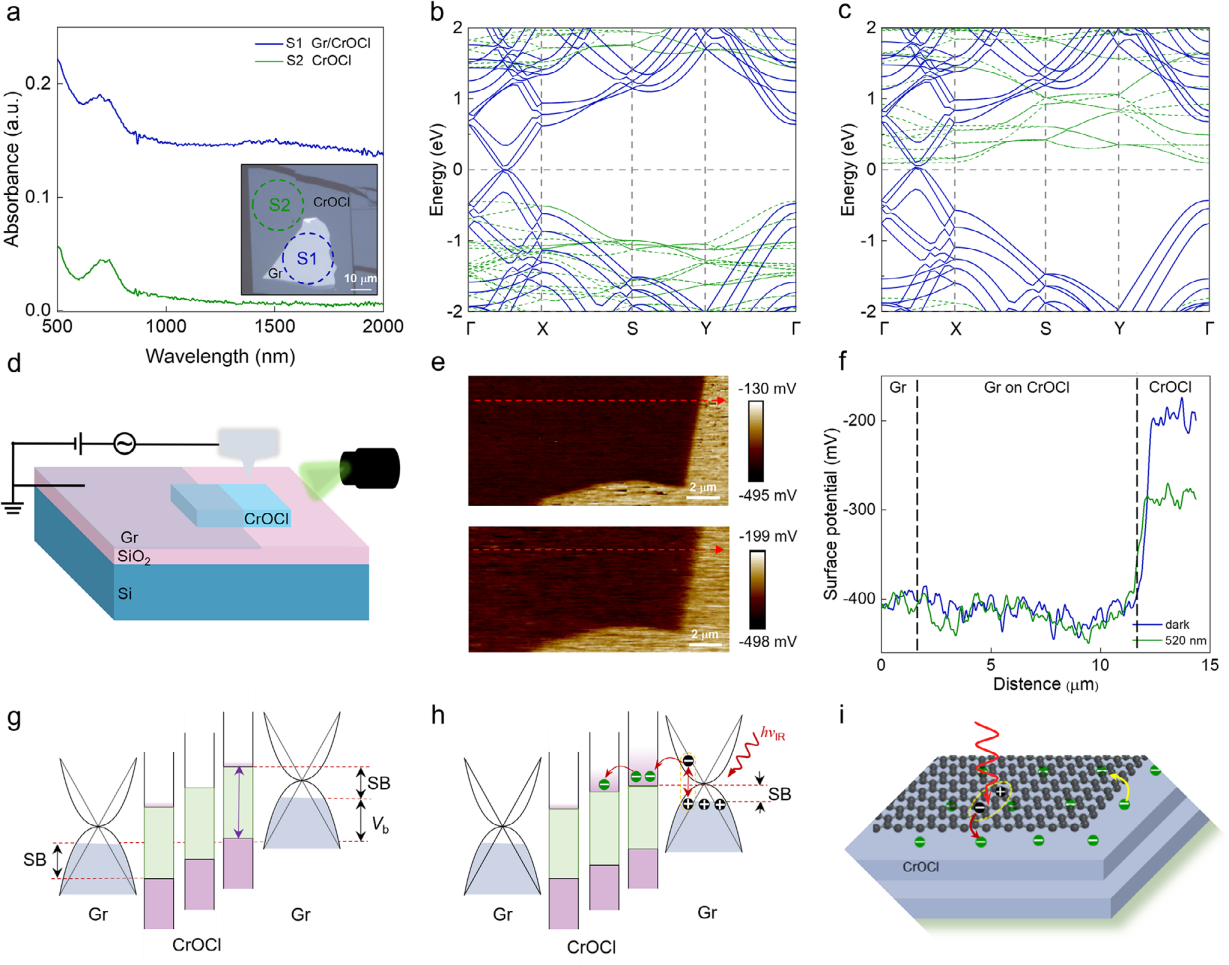
Mechanism of the broadband synaptic response. a) Absorbance spectra of graphene/CrOCl (S1) and CrOCl (S2). Inset is the optical image of the samples. b,c) Calculated band structures of the graphene/CrOCl vdW heterostructure before (b) and after (c) charge transfer from graphene to CrOCl. The conduction band of CrOCl (green dashed lines) is lowered after the interlayer charge transfer and electron doping. d) Schematic diagram of the graphene/CrOCl heterojunction used for KPFM testing. e) Surface potential images of the graphene/CrOCl heterostructure in the dark (top) and upon illumination with light of 520 nm (bottom). f) Surface potential differences derived from (e). g,h) The schematic band alignments of the device before (g) and after (h) IR light illumination. The photo‐generated electrons can transfer to the CrOCl layer, resulting in the decrease of the Schottky barrier. i) Schematic of the graphene/CrOCl, showing the charges transfer between graphene and CrOCl.

The broadband synaptic response in our device originates from the strong interfacial coupling and charge transfer in the graphene/CrOCl vdW heterostructure. Due to its intrinsic zero band gap, graphene is widely utilized in IR detectors.^[^ ^19^, ^39^ ^]^ We compared the absorbance spectra of CrOCl and graphene/CrOCl heterostructure and found that IR light is majorly absorbed by the graphene (shown in **Figure** **4**a). Moreover, the low conductivity in the Au/CrOCl/Au devices suggests that graphene plays a crucial role in the IR optical synaptic response (see Figure S3, Supporting Information). Photogenerated electrons in graphene tend to transfer to the adjacent CrOCl layer due to the strong interfacial coupling. Electrons trapped within the CrOCl form a long‐range Coulomb superlattice^[^ ^24^, ^25^, ^40^ ^]^ and do not contribute to the device conductance. We performed density functional theory (DFT) calculations to compare the band structures of graphene/CrOCl vdW heterostructure before and after electron charge transfer. The calculation results show that electron doping from graphene to CrOCl shifts down the conduction band of the adjacent CrOCl layer (see the green lines in Figure 4b,c; Figure S15, Supporting Information), indicating that charge transfer at the vdW interface is the primary mechanism for modulating the EPSC behavior.

To study the mechanism of IR synaptic photoresponse, we employed Kelvin probe force microscopy (KPFM) to probe the surface potentials of the graphene/CrOCl vdW heterostructure. A vdW heterostructure of graphene/CrOCl was used for KPFM testing in Figure 4d (see optical image and surface morphology in Figure S16, Supporting Information). Figure 4e shows the KPFM images under dark and 520 nm light illuminations, where the surface potentials of graphene, graphene/CrOCl, and CrOCl can be evaluated. Figure 4f is the plots following the dashed arrow lines in Figure 4e, showing that the work function of CrOCl is lowered under 520 nm illumination. The light‐tunable surface potential indicates that light illumination can modulate the tunneling barrier of our device, which makes the electrons easily transfer from graphene to the adjacent CrOCl layer. Notably, the surface potential of graphene located above CrOCl shows a slight decrease compared to the graphene above SiO_2_, hinting the strong interlayer coupling at the vdW interface. For comparison, we fabricated a graphene/h‐BN/graphene device to replace the CrOCl with a thin *h*‐BN flake (Figure S17, Supporting Information), and the device exhibits typical photo response under the illumination of 520 nm laser. The absence of the synaptic behavior in the graphene/h‐BN/graphene device signifies the key role of the interlayer coupling at the CrOCl/graphene interface.

We present a band model to elucidate the EPSC behavior in our FLG/CrOCl/FLG tunneling device before and after IR illumination (shown in Figure 4g,h). Initially, IR light generates photogenerated electron‐hole pairs in the few‐layer graphene layer. Then, electrons from the top graphene layer are transferred to the adjacent CrOCl layer due to strong interface coupling, which lowers the tunneling barrier and increases the tunneling current. Upon the removal of light illumination, electrons in the long‐range charge order gradually retreat from the long‐range superlattice and transfer back to the graphene layer in several minutes (Figure 4i). The formation of the long‐range superlattice by electrons transferred from graphene continuously modulates the tunneling barrier and the EPSC, ultimately leading to the device's broadband light synaptic plasticity. Therefore, the broadband synaptic plasticity is associated with the interfacial coupling between CrOCl and the graphene layer, and is less affected the CrOCl thickness, making it suitable for subsequent practical applications.

### 2.5 IR Orientation Detection

**Figure 5:**
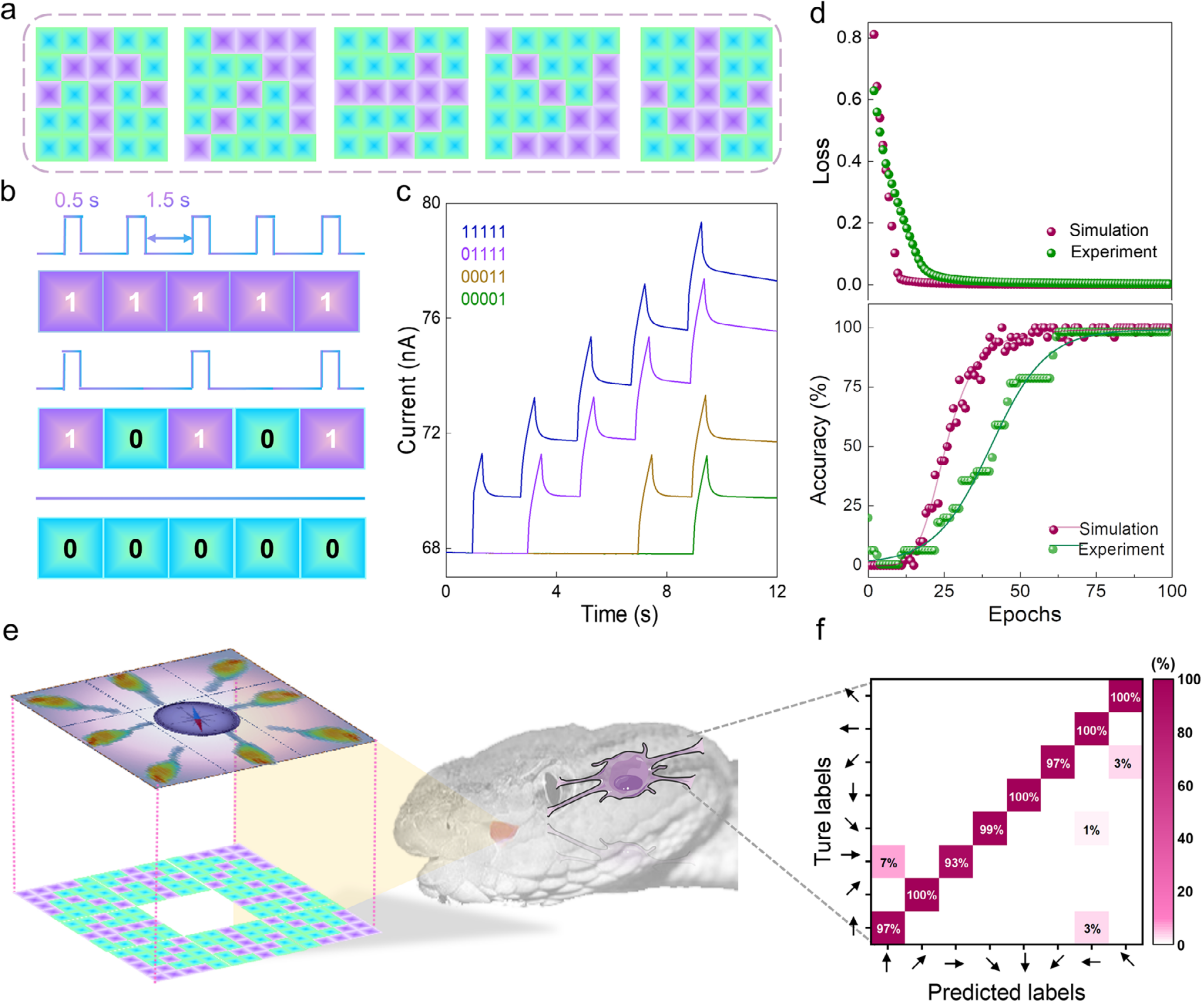
Recognition of images in different orientations based on IR in‐sensor RC system. a) Five images in different orientations used for detection by our device. b) Pulse trains with a binarized number obtained from the orientation of each image in (a). The pulse width is 0.5 s and the pulse interval is 1.5 s. c) *I‐t* photo‐response characteristics of four representative inputs of “00001,” “00011,” “01111,” and “11111”. d) Loss and recognition accuracy of our device for experimental and simulated results with a variation of 0.2. e) 8 mouse images consisting of 23 × 23 pixels mapped to eight different feature directions to mimic the detection process of a pit viper. f) False‐color confusion matrix showing the classification results of our device compared to the predicted output results.

Fast response and real‐time recognition of IR images are crucial for predators hunting in the dark. Due to the broadband synaptic plasticity, our device can achieve feature recognition of IR images, providing valuable insights for simulating various optical synaptic inputs.^[^ ^41^ ^]^ Based on the linear change of the postsynaptic current with the duration of light exposure (Figure S7, Supporting Information), we evaluate the feature recognition capability of the device under 1550 nm laser illumination using five images in different orientations with the RC system.^[^ ^42^ ^]^ The actual images were converted into binary digitized pixels, and each image consists of 25 pixels (shown in **Figure** **5**a) and can be mapped into a pulse train consisting of 5 pulses (Figure 5b). The “ON” and “OFF” states in the pulse train correspond to “1” and “0” in a basic binary image, respectively. Each pulse train applied to our device can obtain an EPSC value, which encodes the information of the orientation (Figure 5c). Based on this translation, all of the images consisting of 25 pixels can be mapped into five bits for further processing (Figure S18, Supporting Information).

The EPSC values were measured using *I‐t* curves for all images (Figure S19, Supporting Information) to assess the detection of orientations. The device demonstrated long‐time stability, capable of withstanding tests for up to 3 months, allowing for ten repetitions per image in both simulation and classification tasks. For the orientation classification, the obtained EPSC values were processed through a virtual network consisting of 5 synapses and 25 weights (the detailed process is shown in Figure S20, Supporting Information). The loss approaches zero after 25 iterations, and the recognition accuracy of 98% is achieved after 60 training epochs for the five directions (shown in Figure 5d), which is comparable to our simulated recognition result with a variation of 0.2.

Furthermore, we map eight distinct mouse images (each consisting of 23 × 23 pixels) to eight different feature directions (shown in Figure 5e) to mimic the brain processing of IR signals recognition by a pit vapor. Gray‐level extraction is performed on the eight images and pulse trains are generated based on rules outlined in Figure 5c. In conjunction with our RC system for orientation classification, we trained 23 × 8 weights and calculated the recognition accuracy. The confusion matrix demonstrates a high recognition accuracy for all eight directions (shown in Figure 5f), indicating the optical synapse based on our FLG/CrOCl/FLG tunneling device is suitable for IR image real‐time recognition, making it promising for future neuromorphic devices and IR vision systems.

## 3 Conclusion

In summary, we reported a few‐layer graphene/CrOCl/few‐layer graphene tunneling device that exhibits optical synaptic characteristics across a broadband range from 520 to 2000 nm. The device converts infrared signals with high efficiency and simulates various synaptic behaviors, including EPSC, STDP, SNDP, SRDP, and PPF. Furthermore, integrated with RC, our device can effectively detect infrared signals and classify the IR images with different orientations. Both experimental and simulation results achieve recognition rates exceeding 98% within 60 epochs, providing valuable insights for the development of multifunctional, intelligent perception systems. Consequently, this vdW heterostructure holds significant promise for advancing optoelectronic synaptic devices and enhancing the performance of IR recognition systems.

## 4 Experimental Section

### h‐BN/ Graphene/CrOCl/ Graphene Heterojunction Fabrication

The *h*‐BN/ graphene/CrOCl/ graphene heterojunction was fabricated using a dry transfer technique. All materials were mechanically exfoliated using 3 M scotch tape on flake blocks onto a silicon/silicon dioxide (285 nm) (Si/SiO_2_) substrate for optical microscopy examination (Olympus). First, a poly (Bisphenol A carbonate) (PC) film coated with polydimethylsiloxane (PDMS) was used to pick up *h*‐BN. Next, top graphene and CrOCl were sequentially picked up using the strong vdW force between *h*‐BN and 2D materials. The PC was prepared by dispersing 1 g of polycaprolactone in 10 mL chloroform (Aladdin Tech. Co.). Then, the picked‐up *h*‐BN, top graphene, and CrOCl were aligned and released onto the bottom graphene at 180 °C. Finally, the PC was removed by soaking in chloroform for 5 min. Use electron‐beam lithography technology (Zeiss supra 55 and Raith ELPHY Quantum) to pattern the electrons of the device, and deposit Cr/Au (5/50 nm) with thermal evaporation in a high vacuum of 10^−5^ Pa by a thermal evaporation coater (Chinese Academy of Sciences Shenyang Scientific Instruments Co.).

### Au/CrOCl/Au Device Fabrication

The mechanically exfoliated CrOCl was picked up from the substrate using a PC and released onto the pre‐deposited bottom electrode Cr/Au (5/10 nm) at 180 °C. Then, the prepared sample was evaporated the top electrode Cr/Au (5/50 nm) in the region where CrOCl overlaps with the bottom electrode.

### Characterization of Tunneling Devices

The thicknesses of materials were performed using atomic force microscopy (Dimension Icon, Bruker). Absorption spectra were obtained by a UV–Vis‐NIR Microscopic Spectrophotometer (MSV‐5700, Jasco). The TEM images were obtained by an FEI Themis Z with double aberration correctors, and the data was processed by the Velox software.

### Photoelectronic Measurement

Photoelectric measurements were performed in a probe (CRX‐6.5K, Lakeshore) using a semiconductor analyzer (Keysight B1500A). 520, 1064, and 1550 nm laser (NBeT) were introduced directly into the probe shore cavity through an optical fiber and the light intensity was measured by a power meter (THORLABS GmbH). Lasers with other wavelengths were introduced into the probe cavity directly through optical fibers via a continuous‐wave laser.

### KPFM Measurement

The KPFM was based on the Bruker by a conducting AFM tip coated with Pt/Ir under ambient conditions. Before performing real measurements of the samples, the instrument was calibrated using an Au electrode deposited on a SiO_2_/Si substrate. The KPFM measurements of the sample (graphene/CrOCl heterojunction) performed in the dark and under 520 nm laser illumination used the same settings without lifting the tip or changing any parameters. Based on the levels of work functions in graphene and CrOCl, the band diagrams of this heterostructure in the dark and under 520 nm laser illumination were estimated and depicted.

### First‐Principles Calculation

The density functional theory (DFT) calculations were performed by using the Vienna ab initio simulation package (VASP).^[^ ^43^, ^44^ ^]^ The core electrons were described with the projector augmented wave method. The exchange correlation potential adopts the generalized gradient approximation (GGA) in the Perdew–Burke–Ernzerhof form.^[^ ^45^ ^]^ The GGA + U approach^[^ ^46^, ^47^ ^]^ was adopted with a value of U = 2.7 eV for the Cr‐3d electron, which had been tested in previous works.^[^ ^40^ ^]^ The heterostructure comprises Bernal (ABA) stacked trilayer graphene and monolayer CrOCl. The lattice constant for graphene was 2.46 Å, and the lattice constants of CrOCl in the a and b directions were 3.24 and 3.93 Å, respectively. A computational supercell of the heterostructure contains a 4 × 2√3 × 1 supercell of trilayer graphene and a 3 × 2 × 1 supercell of monolayer CrOCl, resulting in a lattice mismatch of 1.2% and 0.66 for graphene in the a and b directions, respectively. Due to the different electronegativity, there exists electron transfer from graphene to the CrOCl substrate. Besides, the photoelectrons generated on graphene and transferred to the substrate strengthen the non‐equilibrium charge distribution. To capture this point, the electronic energy bands of trilayer graphene doped with 0.1 hole doping and that of CrOCl under 0.1 electron doping were calculated. The energy cutoff for the plane wave basis was set to be 500 eV, and a 4 × 6 × 1 k‐point mesh was used to sample the first Brillouin zone. The convergence thresholds of energy and force were set to be 10^−6^ eV and 10^−2^ eV Å^−2^, respectively. A vacuum of 15 Å was introduced to avoid the interaction between periodic images.

## Conflict of Interest

The authors declare no conflict of interest.

## Author Contributions

D.G conducted the experimental test and wrote the manuscript. W.L. and F.Z. conducted the theoretical calculations. P.G. and Y.Y. synthesized CrOCl samples. W.D. finished the TEM test. X.R. assisted in the test of all optical synapses. K.W. and T.T. provided hBN samples. S.Z. and J. Z. supervised the project. All authors participated in discussions and contributed to the manuscript.

## Supporting information



Supporting Information

## Data Availability

The data that support the findings of this study are available from the corresponding author upon reasonable request.
