# Supplementary material for: A Van Der Waals Broadband Infrared Optical Synapse Enabling Orientation Detection
Source: Adv Sci (Weinh). 2025 Sep 30;12(48):e07530. doi: 10.1002/advs.202507530 (PMC12752591; doi:10.1002/advs.202507530)
Supplement: Supplementary file 1 — Supporting Information [file ADVS-12-e07530-s001.docx]

**Supplementary Information**

**A Van Der Waals Broadband Infrared Optical Synapse
Enabling Orientation Detection**

*Dan Guo^1^, Wenjing Li^1^, Pingfan Gu^2^，Weikang Dong^1^, Xuyan Rui^1^, Kenji Watanabe^3^, Takashi Taniguchi^3^, Yu Ye^2^, Fawei Zheng^1^, Jiadong Zhou^1,4,^*, Shoujun Zheng^1,4,^**

*^1^Centre for Quantum Physics, Key Laboratory of Advanced Optoelectronic Quantum Architecture and Measurement (MOE), School of Physics, Beijing Institute of Technology, Beijing, 100081, China*

*^2^State Key Laboratory for Artiﬁcial Microstructure & Mesoscopic Physics and Frontiers Science Center for Nano-Optoelectronics School of Physics, Peking University, Beijing 100871, China*

*^3^National Institute for Materials Science, 1-1 Namiki, Tsukuba 303-0044, Japan*

*^4^Faculty of Marine Science and Technology, Beijing Institute of Technology, Zhuhai, Guangdong, 519088, China*

*Corresponding email: jdzhou@bit.edu.cn,* [*szheng@bit.edu.cn*](mailto:szheng@bit.edu.cn)


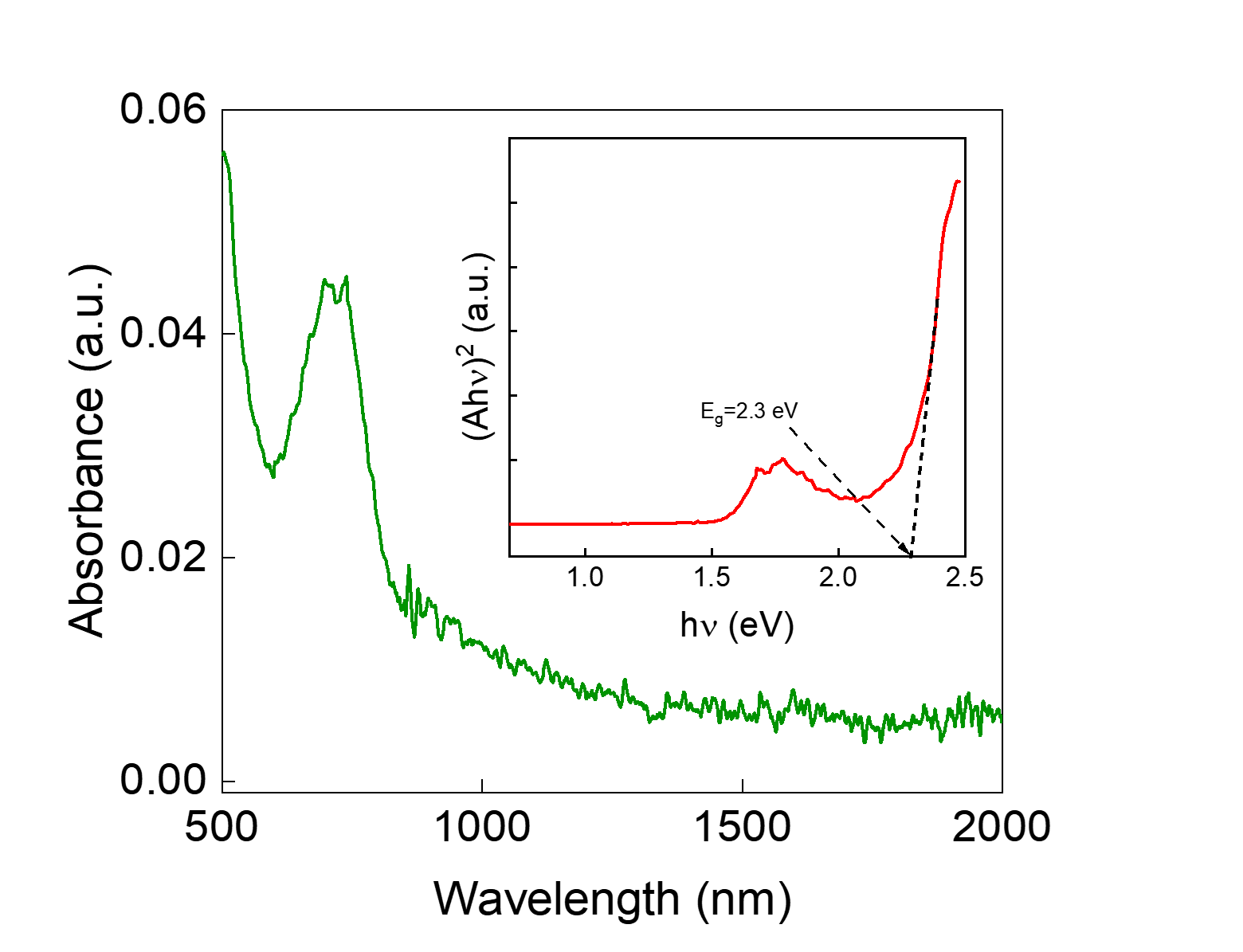


**Figure S1.** **The ultraviolet-visible-infrared absorption spectra of CrOCl. The inset is the corresponding Tauc plot, showing a bandgap of 2.3 eV.**

**
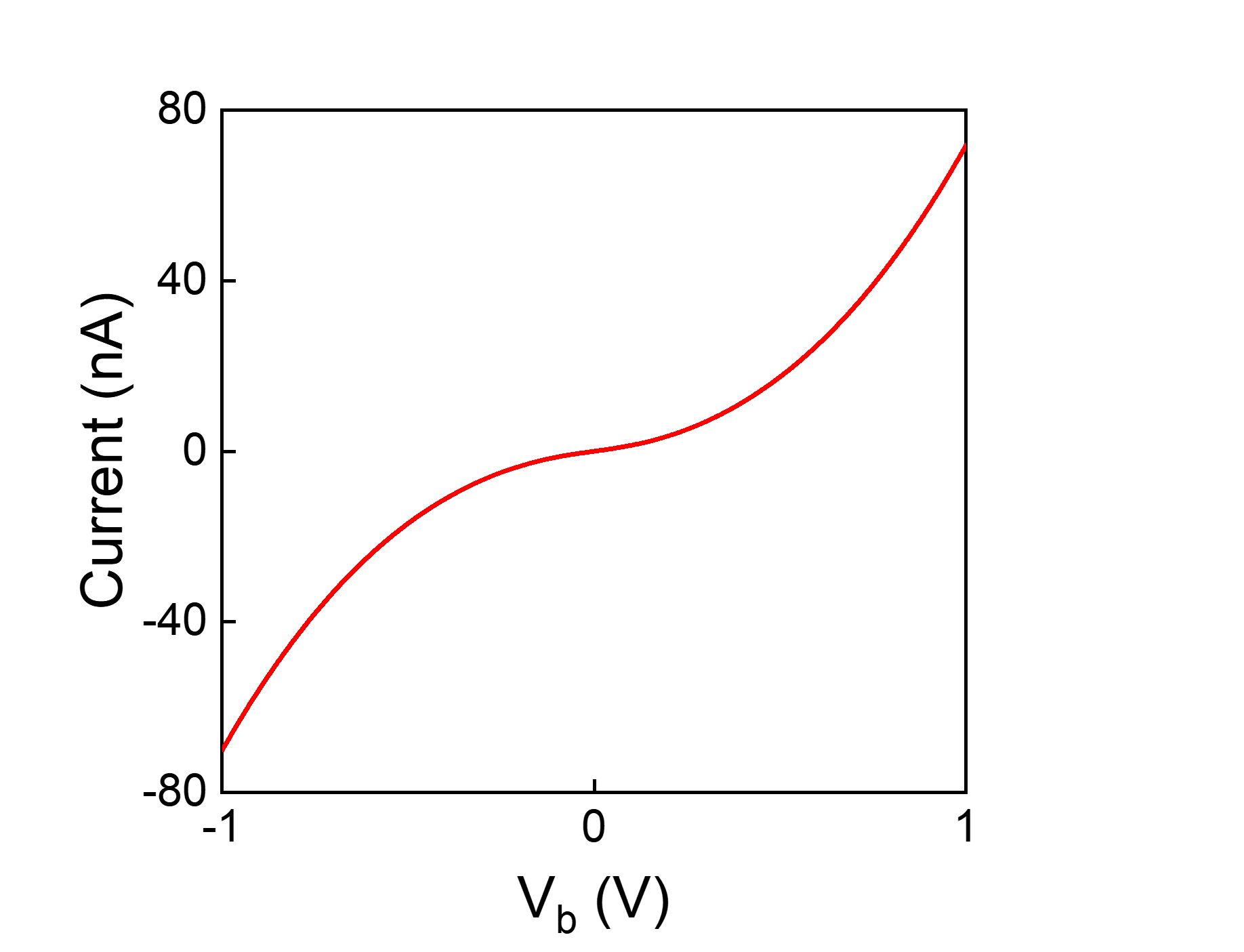
**

**Figure S2.** **Output characteristic of the device in the dark with V_g_=0 V.**


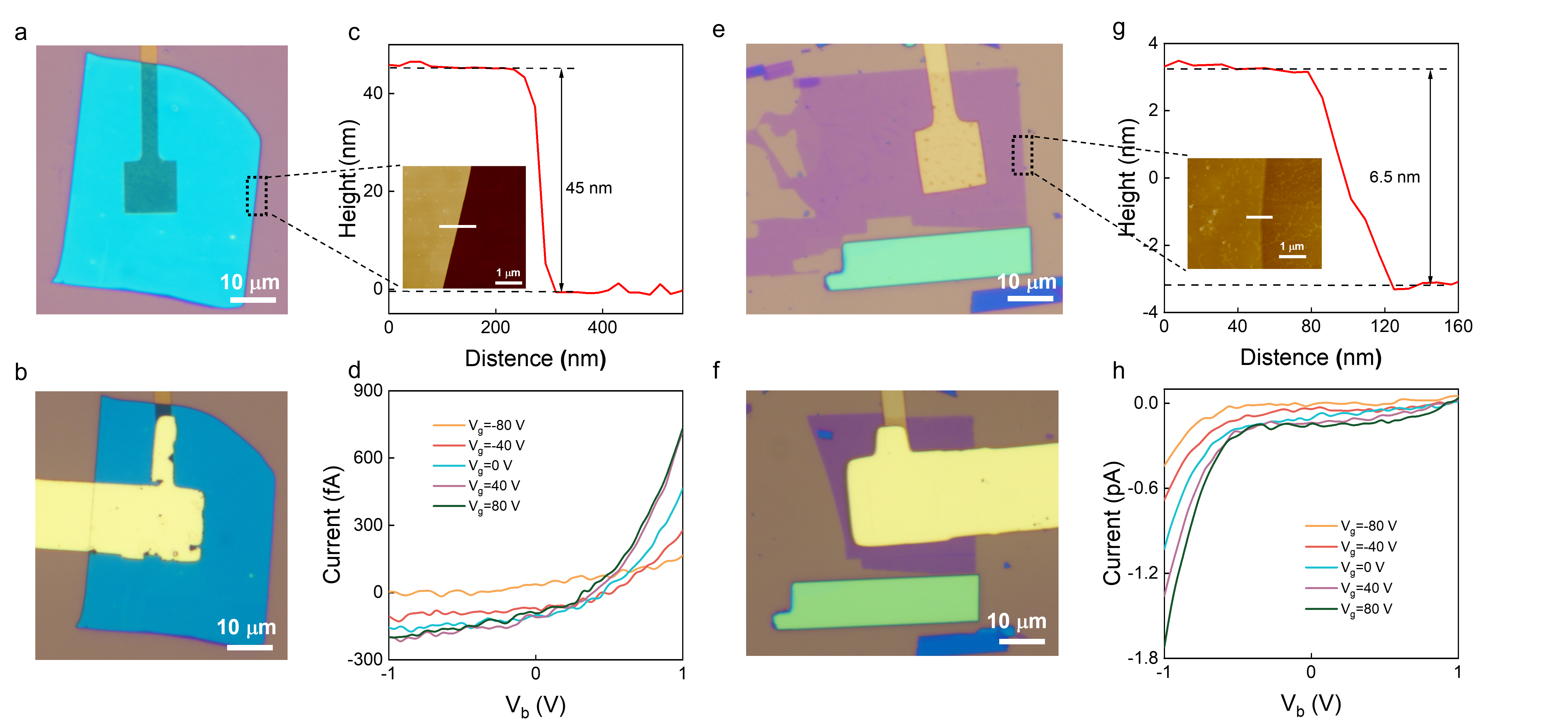


**Figure S3.** **Characterizations of Au/CrOCl/Au device with the CrOCl thickness of 45 nm (a-d) and 6.5 nm (e-h).** (a, e) Optical images of Au-CrOCl. (b, f) Optical images of Au-CrOCl-Au device. (c, g) AFM characterizations of CrOCl thickness. Insets show the morphology of CrOCl within the dashed box in (a) and (e), respectively. (d, h) Output characteristic curves of the Au-CrOCl-Au device, showing the excellent insulating behavior of CrOCl.

**
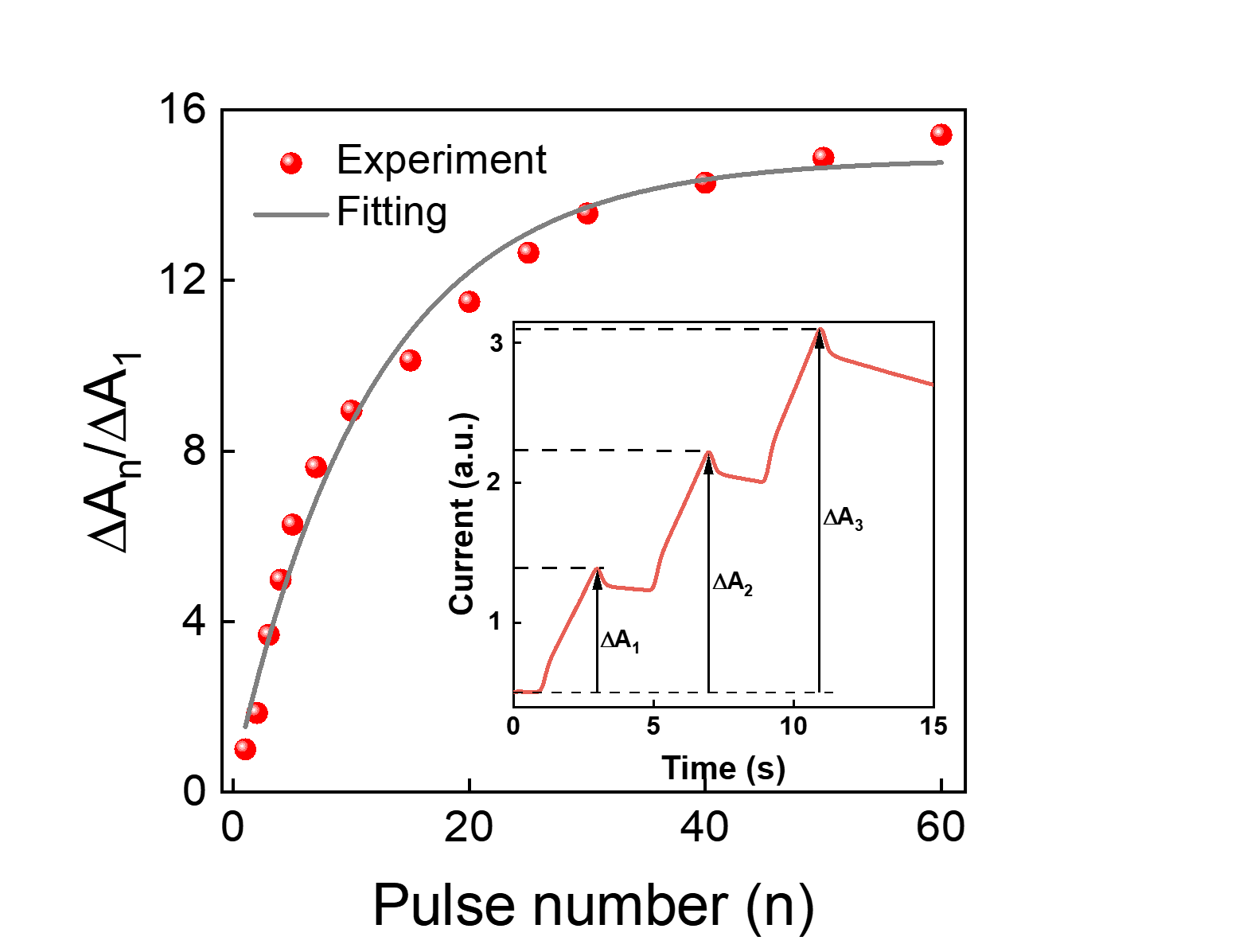
**

**Figure S4. ΔA_n_/ΔA_1_ of the device as a function of pulse number of 1550 nm at V_g_=0 V and V_b_=1 V with the duration of a single optical pulse is 2 s and the power of 51 μW. Inset shows the simulated behavior of three light pulses.**

**
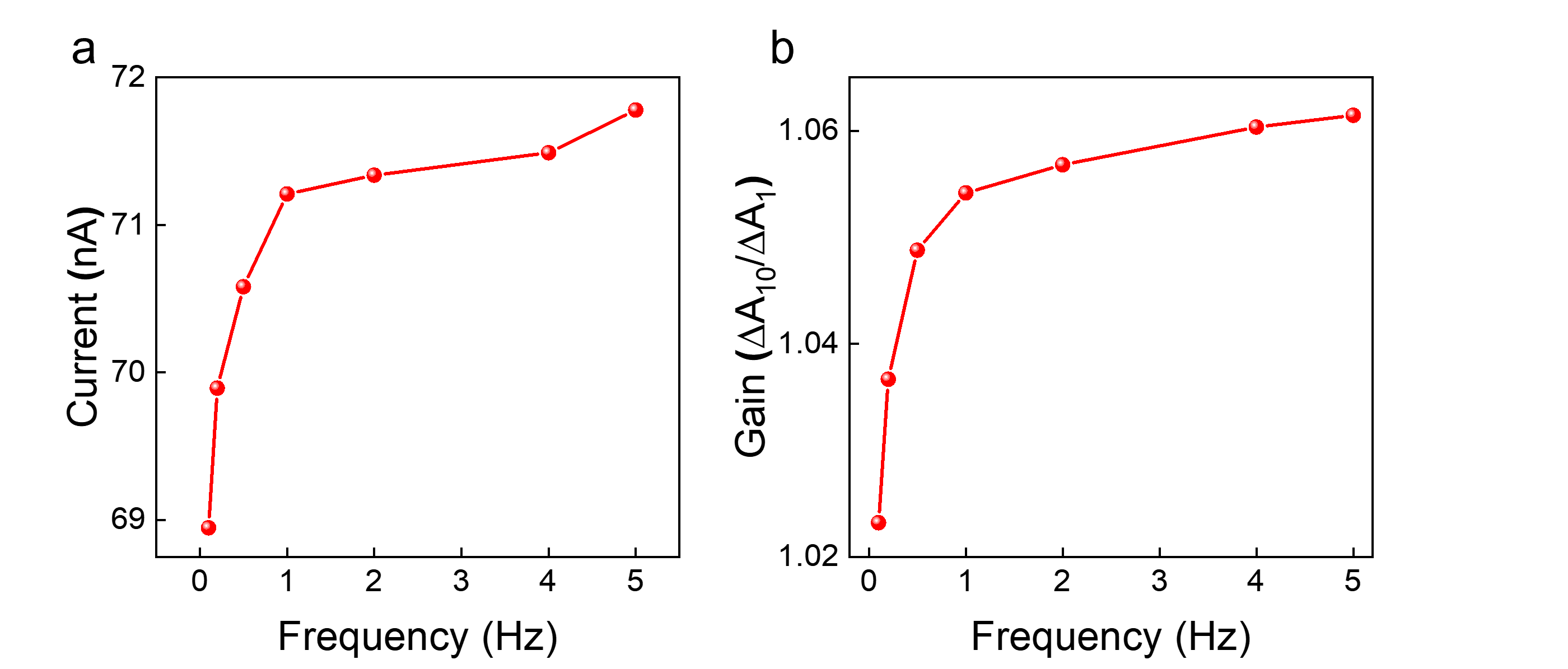
**

**Figure S5.** **The frequency dependence of the device under 1550 nm laser pulse about 0.1 s with 51 µW at V_g_=0 V and V_b_=1 V.** (a) Current of the device stimulated by 10 optical spikes at the spiking frequencies from 0.1 to 5 Hz. (b) Dependence of gain (ΔA_10_/ΔA_1_) on the spiking frequency. A_10_ and A_1_ are the maximum values of the current evoked by the tenth optical spike and that evoked by the first optical spike, respectively.

**
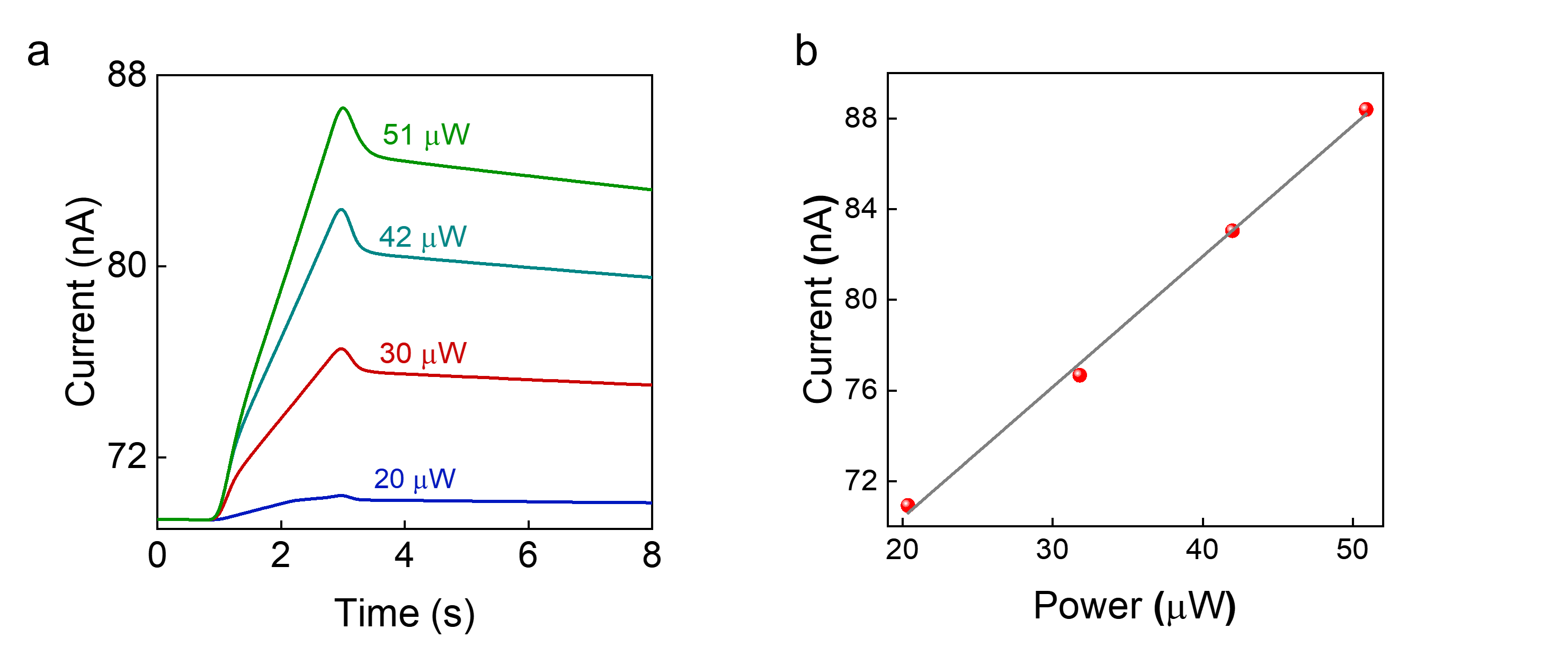
**

**Figure S6. The laser power dependence of the device under 1550 nm laser pulse at V_g_=0 V and V_b_=1 V.** (a) Power-dependent photoresponse with the optical pulse of 2 s. (b) The current value of the device after a 2 s light pulse of 1550 nm as a function of light power, showing an approximately linear relationship between photocurrent and laser power. (The gray line is the fit of the current values.)

**
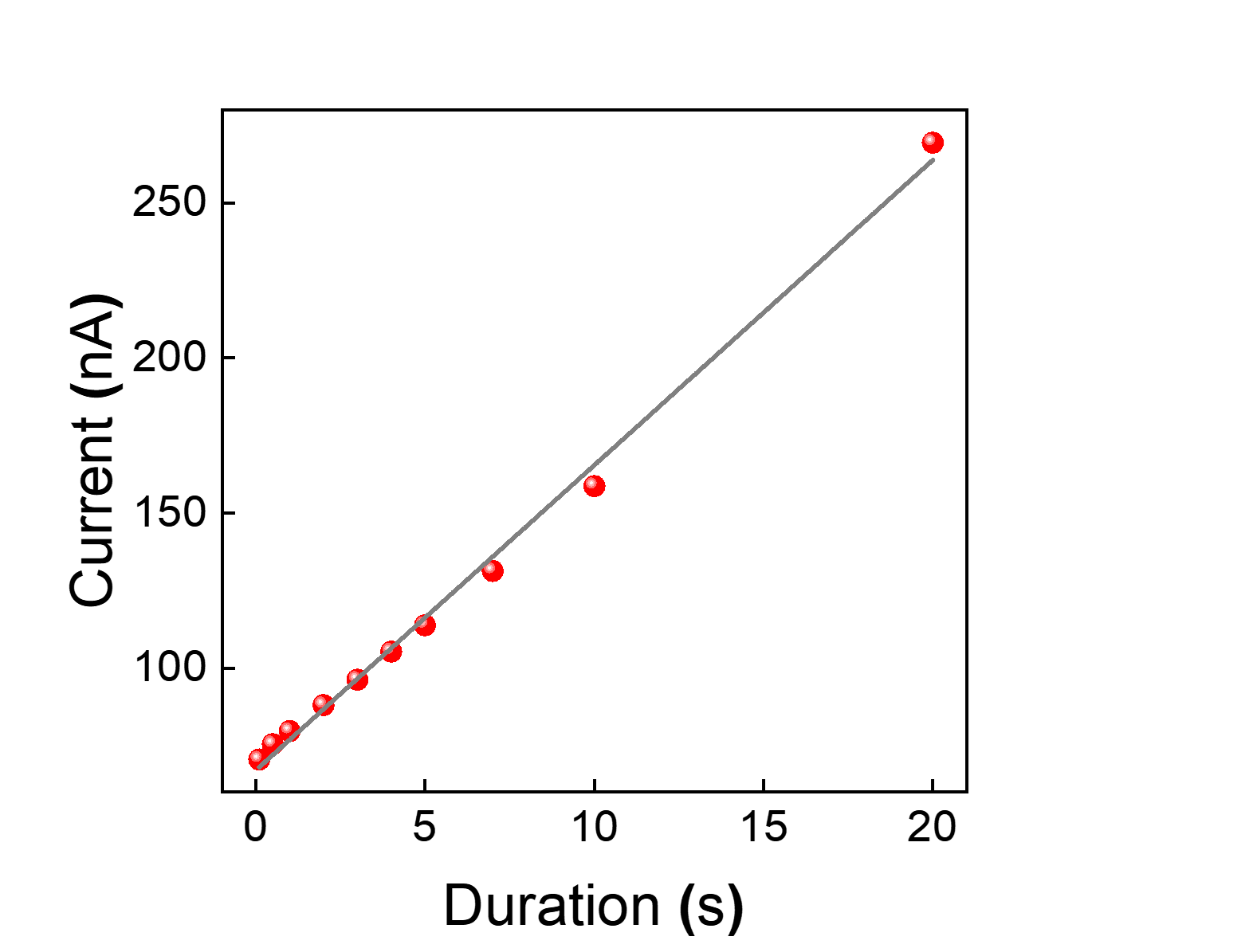
**

**Figure S7. The current value of the device after different light pulse duration of 1550 nm at 51 uW with V_g_=0 V and V_b_=1 V, showing an approximately linear relationship between photocurrent and laser duration. (The gray line is the fit of the current values.)**

**
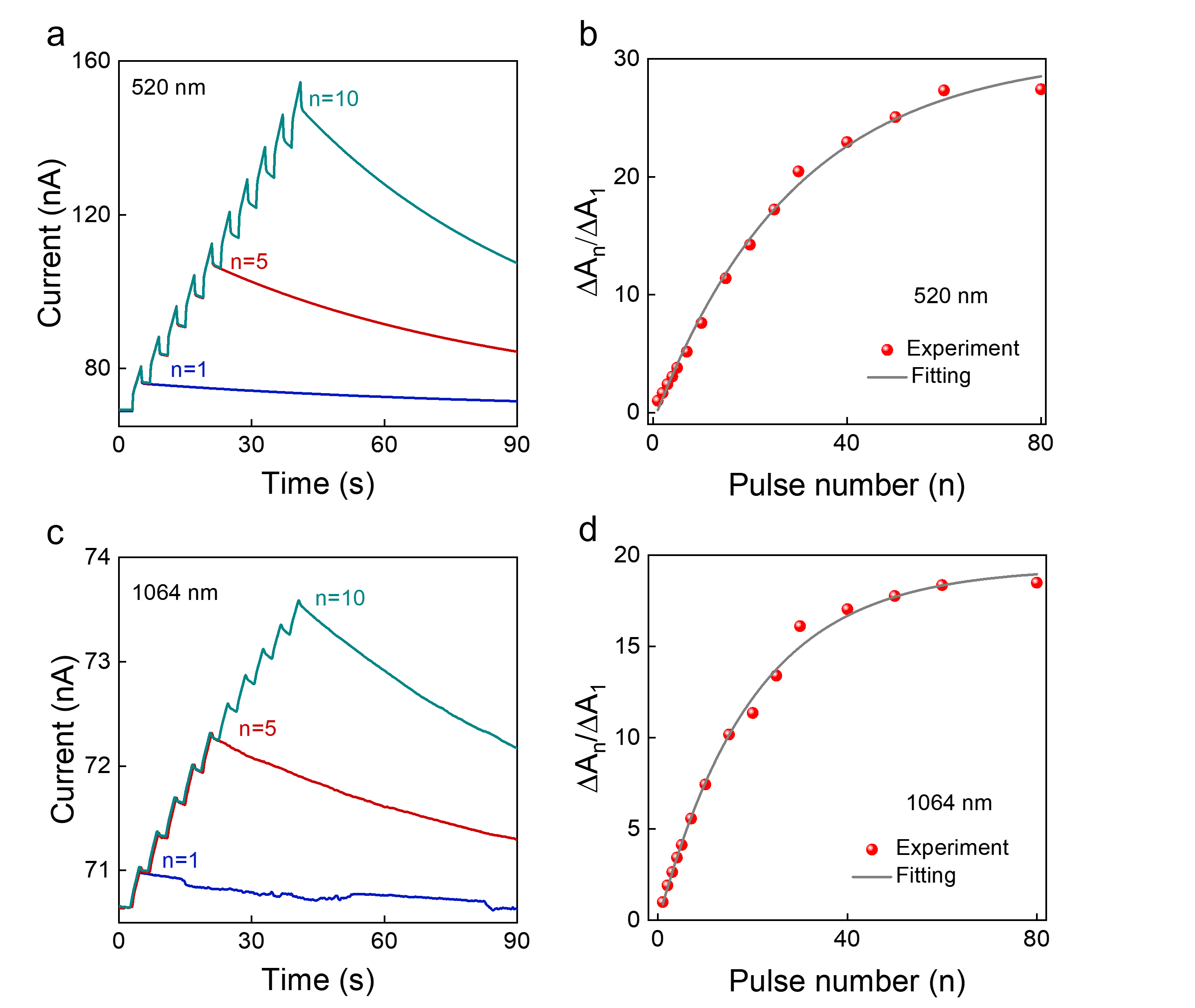
**

**Figure S8. The optical synaptic behavior of the device under 520 nm laser of 38 uW (a, b) and 1064 nm laser of 44 uW (c, d) with the duration of a single optical pulse is 2 s at V_g_=0 V and V_b_=1 V.** (a, c) SNDP triggered by diﬀerent numbers of optical pulses about 520 nm (a) and 1064 nm (c) laser. (b, d) ΔA_n_/ΔA_1_ of the device as a function of the number of applied optical pulses about 520 nm (b) and 1064 nm (d) laser.

**
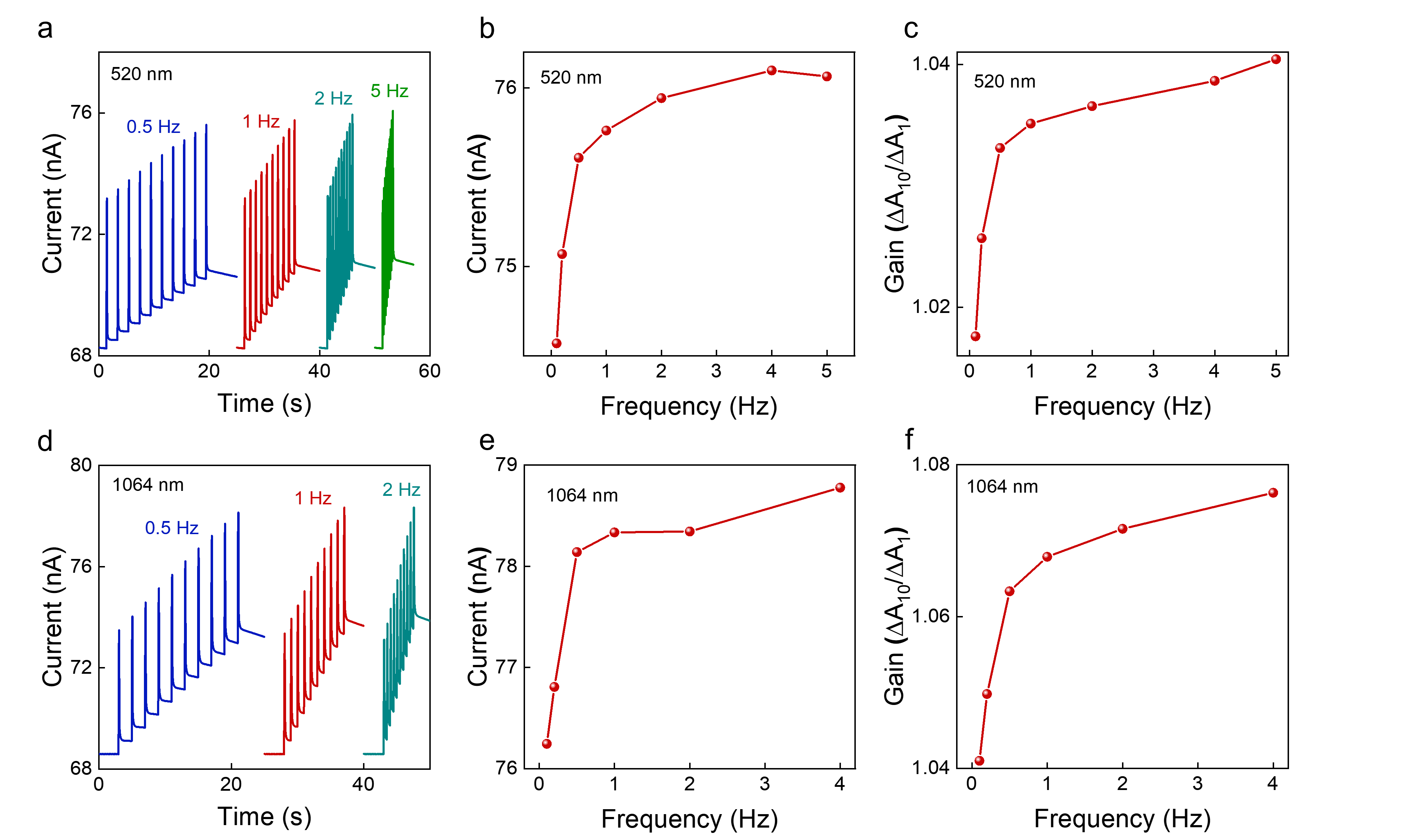
**

**Figure S9. The frequency dependence of the device under 520 nm laser pulse with 38 µW (a-c) and 1064 nm laser pulse with 44 uW (d-f) about light pulse of 0.1 s at V_g_=0 V and V_b_=1 V.** (a, d) SRDP triggered by diﬀerent rates of optical pulse about 520 nm (a) and 1064 nm laser (d). (b, e) Current of the device stimulated by 10 optical spikes about 520 nm (b) and 1064 nm laser (e). (c, f) Dependence of gain (ΔA_10_/ΔA_1_) on the spiking frequency about 520 nm (c) and 1064 nm laser (f). A_10_ and A_1_ are the maximum values of the current evoked by the tenth optical spike and that evoked by the first optical spike, respectively.

**
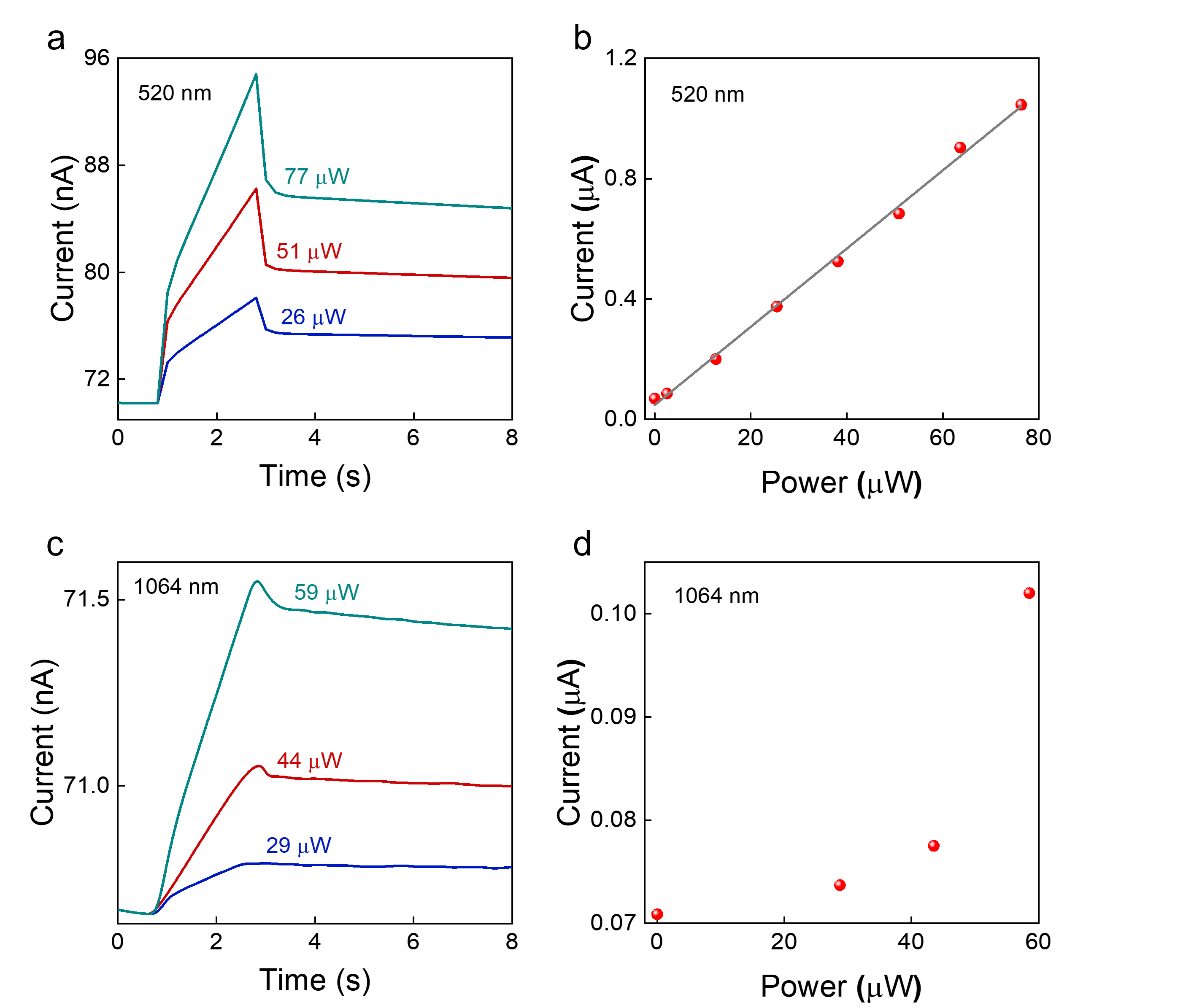
**

**Figure S10. The optical synaptic behavior of the device under 520 nm (a,b) and 1064 nm (c,d) laser with the duration of the optical pulse is 2 s at V_g_=0 V and V_b_=1 V.** (a, c) Light power-dependent photoresponse under 520 nm (a) and 1064 nm (c) laser. (b, d) The current value of the device after light pulse of 520 nm (b) and 1064 nm (d) as a function of light pulse power. (The gray line is the fit of the current values).

**
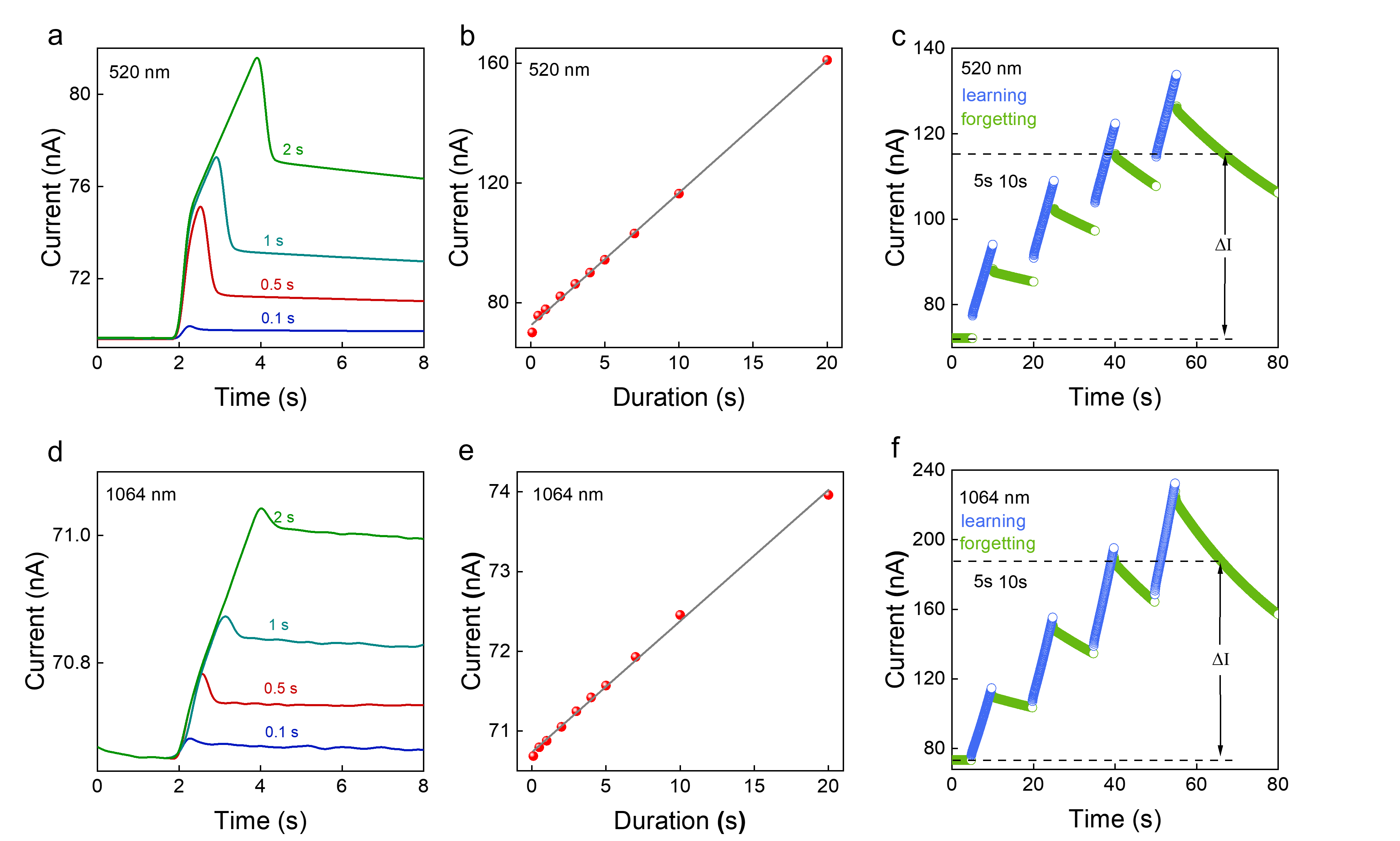
**

**Figure S11. The optical synaptic behavior of the device under 520 nm laser of 38 uW (a-c) and 1064 nm laser of 44 uW (d-f) at V_g_=0 V and V_b_=1 V.** (a, d) Optical pulse duration-dependent photoresponse about 520 nm (a) and 1064 nm (d) laser. (b, e) The current value of the device after different light pulse durations about 520 nm (b) and 1064 nm (e) laser, showing an approximately linear relationship between photocurrent and laser duration. (The gray line is the fit of the current values.) (c, f) The simulated human learning-forgetting-relearning process about 520 nm (c) and 1064 nm (f).

**
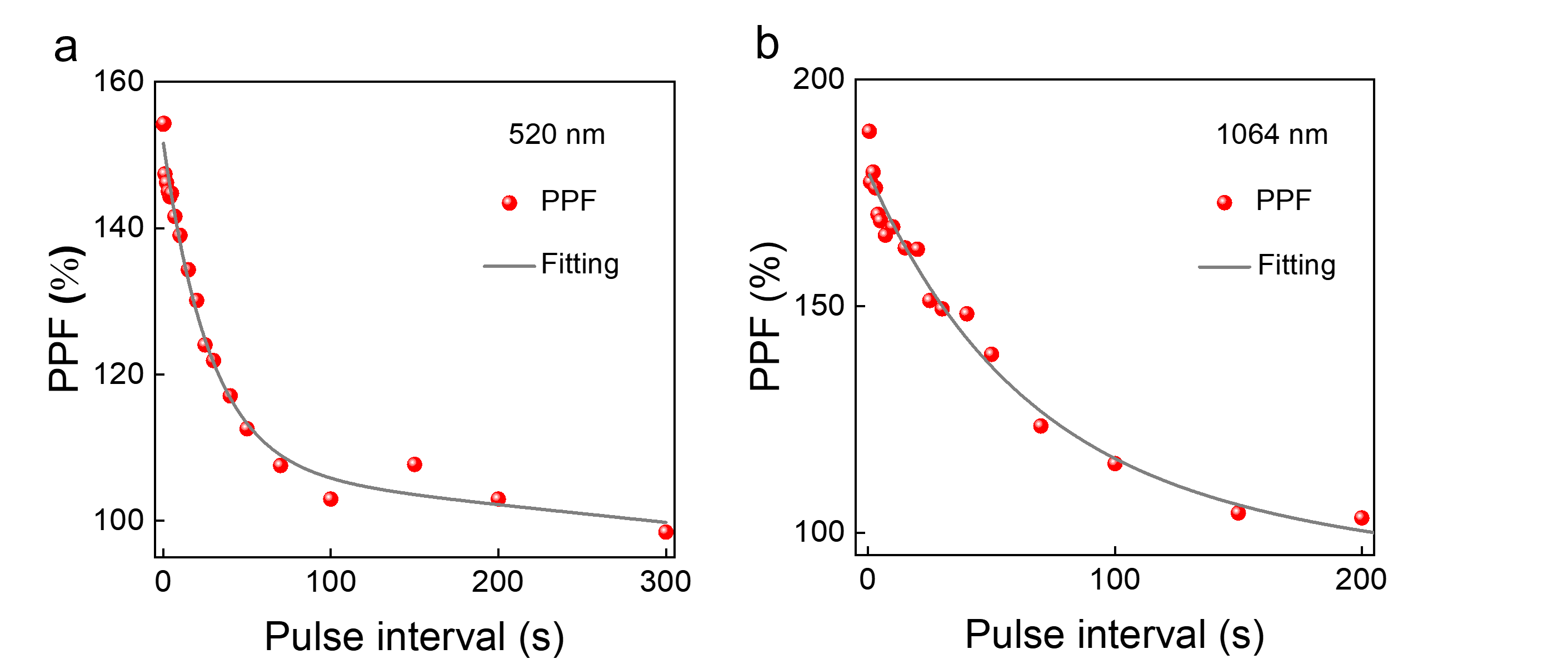
**

**Figure S12. Dependence of the PPF ratio (defined as ΔA_2_/ΔA_1_×100%) on the pulse interval under the power of 38 μW of 520 nm laser (a) and 44 μW of 1064 nm laser (b) with a light pulse of 1 s at V_g_=0 V and V_b_=1 V. Both insets show the PPF behavior stimulated by a pair of optical pulses.**

**
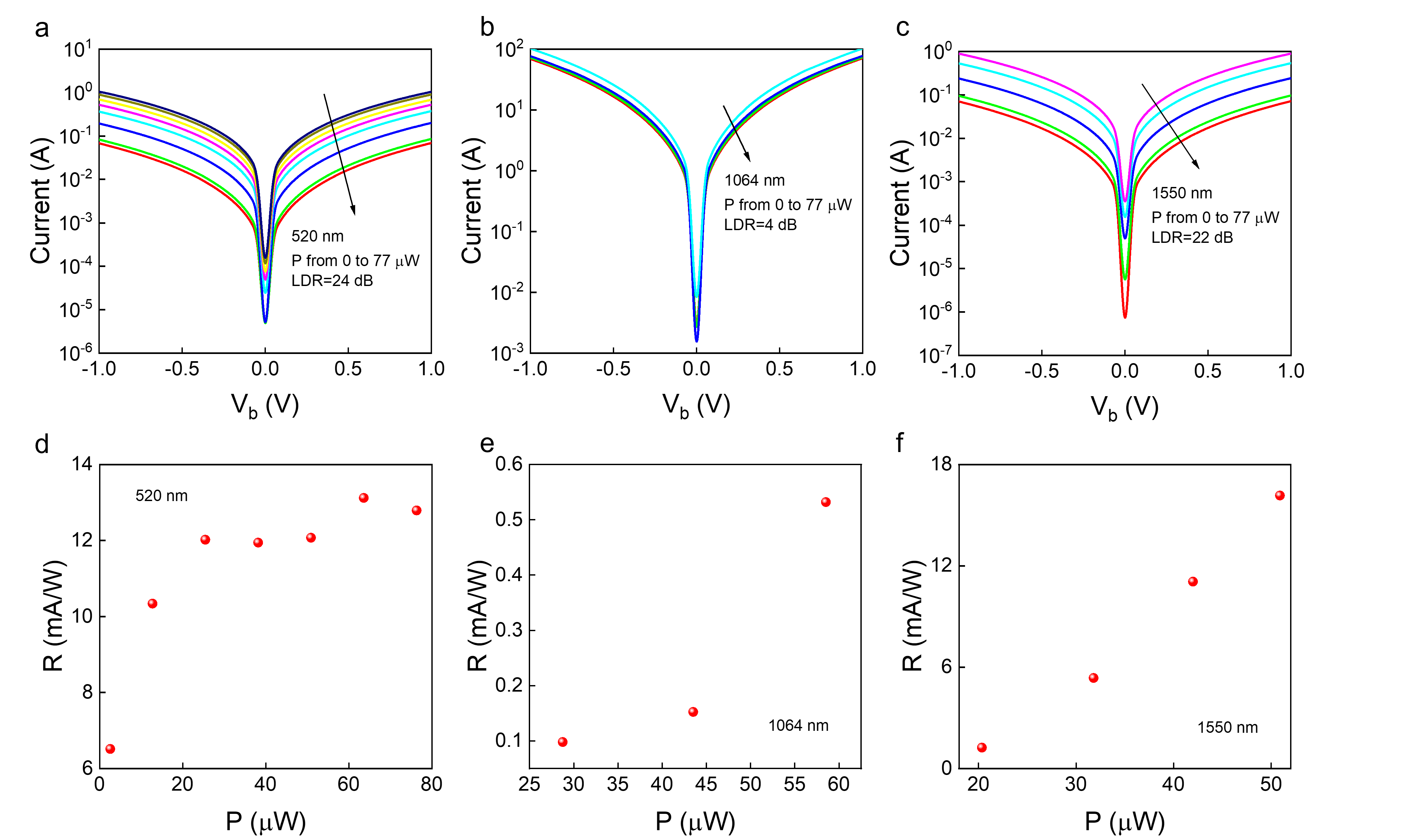
**

**Figure S13.** **Important parameters of light detection capability.** a-c) The photocurrent curves of the device under 520 nm (a), 1064 nm (b), and 1550 nm (c) light illumination, with the calculated dynamic ranges of 24 dB, 4 dB, and 22 dB, respectively. d-f) The responsivity of the device under 520 nm (d), 1064 nm (e), and 1550 nm (f) light illumination.

**
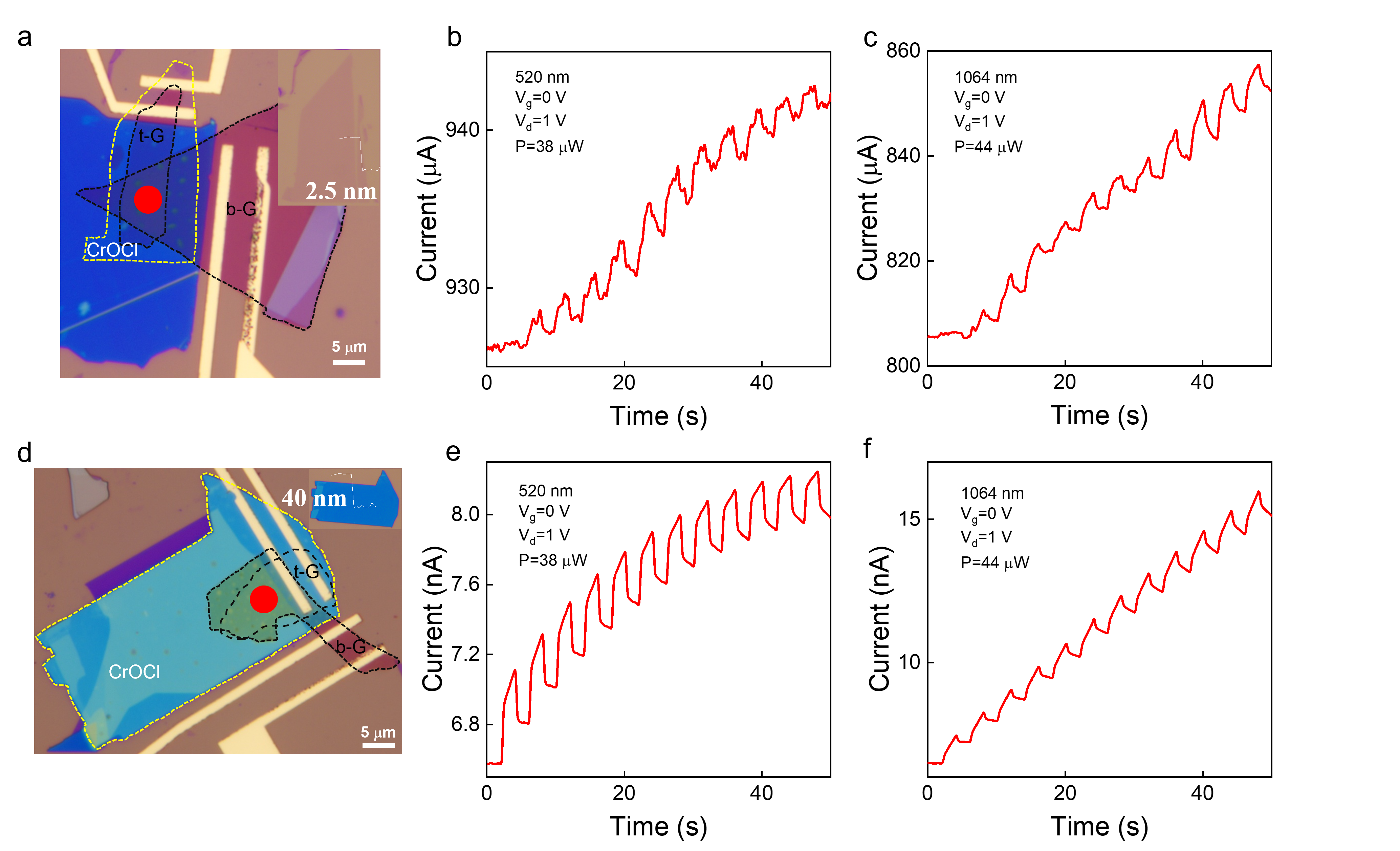
**

**Figure S14. Characterizations of the few-layer graphene/CrOCl/few-layer graphene device with the CrOCl of 2.5 nm (a-c) and 40 nm (d-f).** a,d) Optical image of the device with the CrOCl of 2.5 nm (a) and 40 nm (d), which are fully encapsulated in h-BN. Few-layer graphene and CrOCl are highlighted with black and yellow dashed lines, and the device’s active region is highlighted by the red circle. b,e) Endurance measurement of the photo current at Vg=0 V and Vd=1V of a 38 µW, 520 nm laser, showing pronounced synaptic plasticity. c,f) Endurance measurement of the photo current at Vg=0 V and Vd=1V of a 44 µW, 1064 nm laser, showing pronounced synaptic plasticity.

**
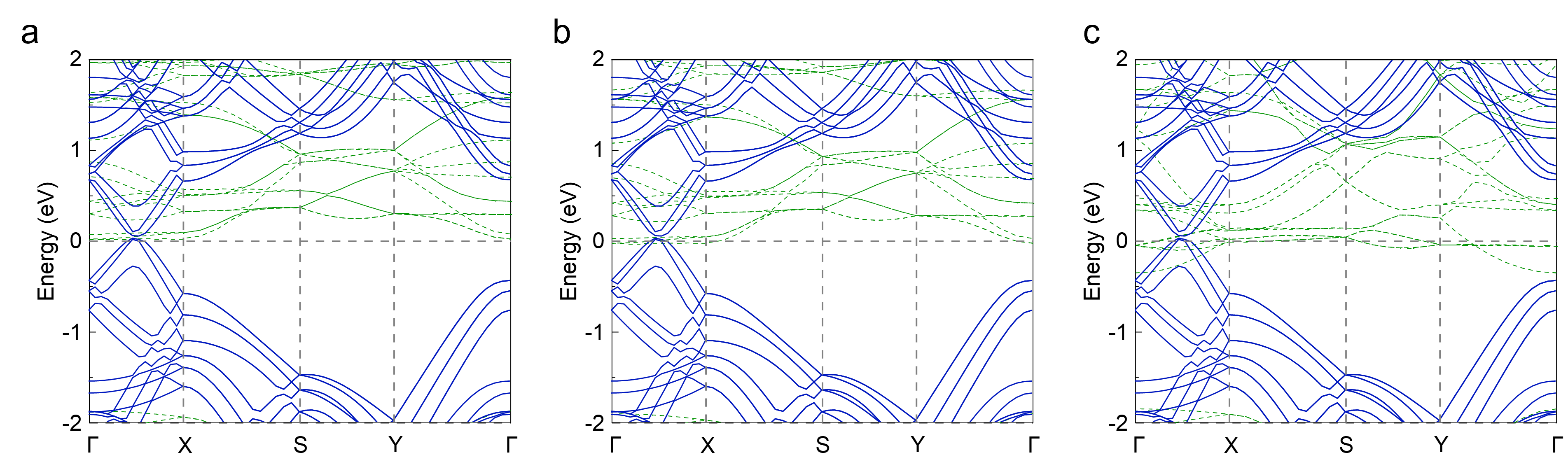
**

**Figure S15. Calculated band structures of the graphene/CrOCl vdW heterostructure after charge transfer from graphene to CrOCl for 0.5 hole/electron doping (a), 1 hole/electron doping (b) and 5 hole/electron doping (c). The conduction band of CrOCl (green dashed lines) is lowered after the interlayer charge transfer and electron doping.**

**
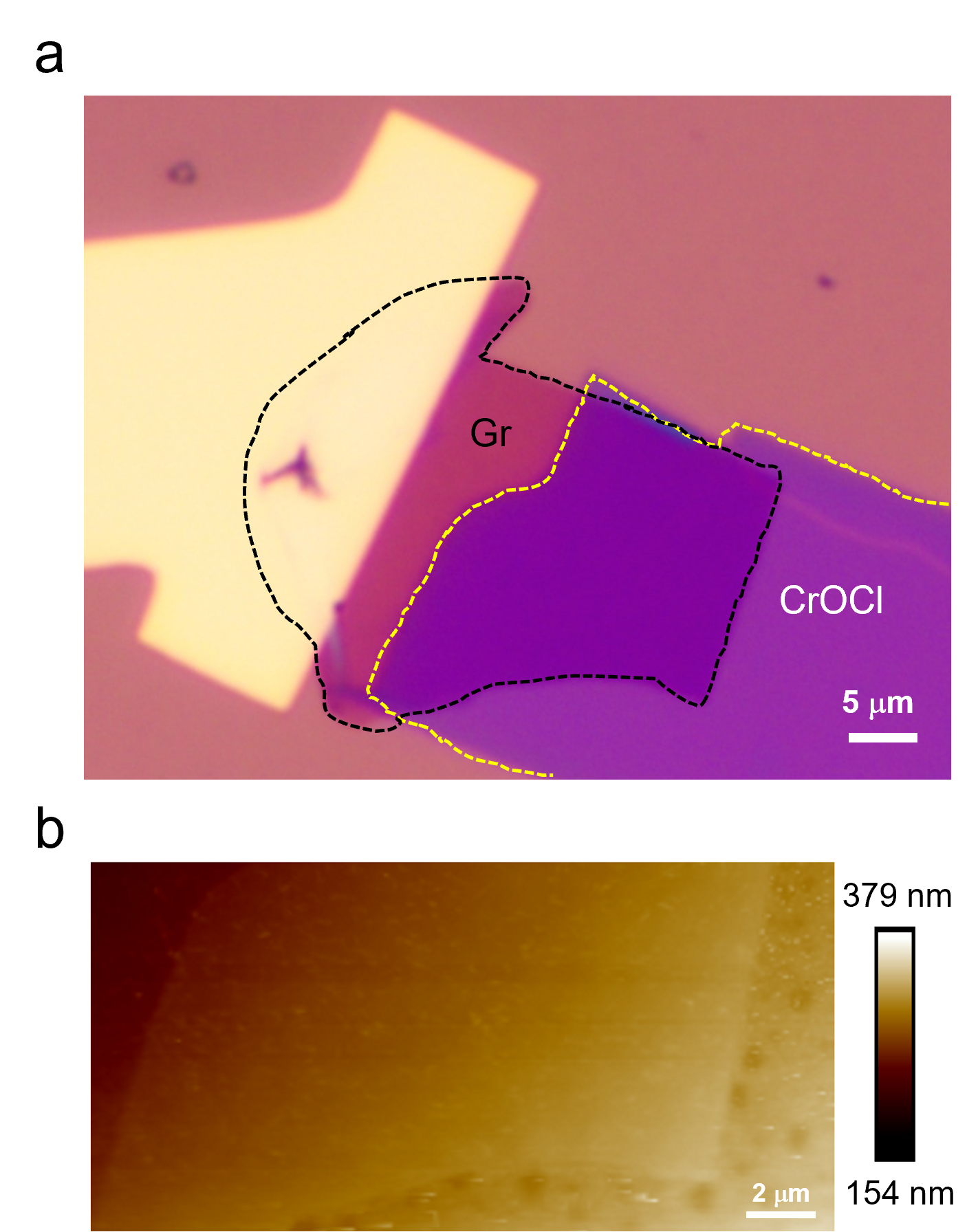
**

**Figure S16.** a) Optical image of the graphene/CrOCl heterojunction used for KPFM testing. b) Surface morphology of the region used for KPFM testing.


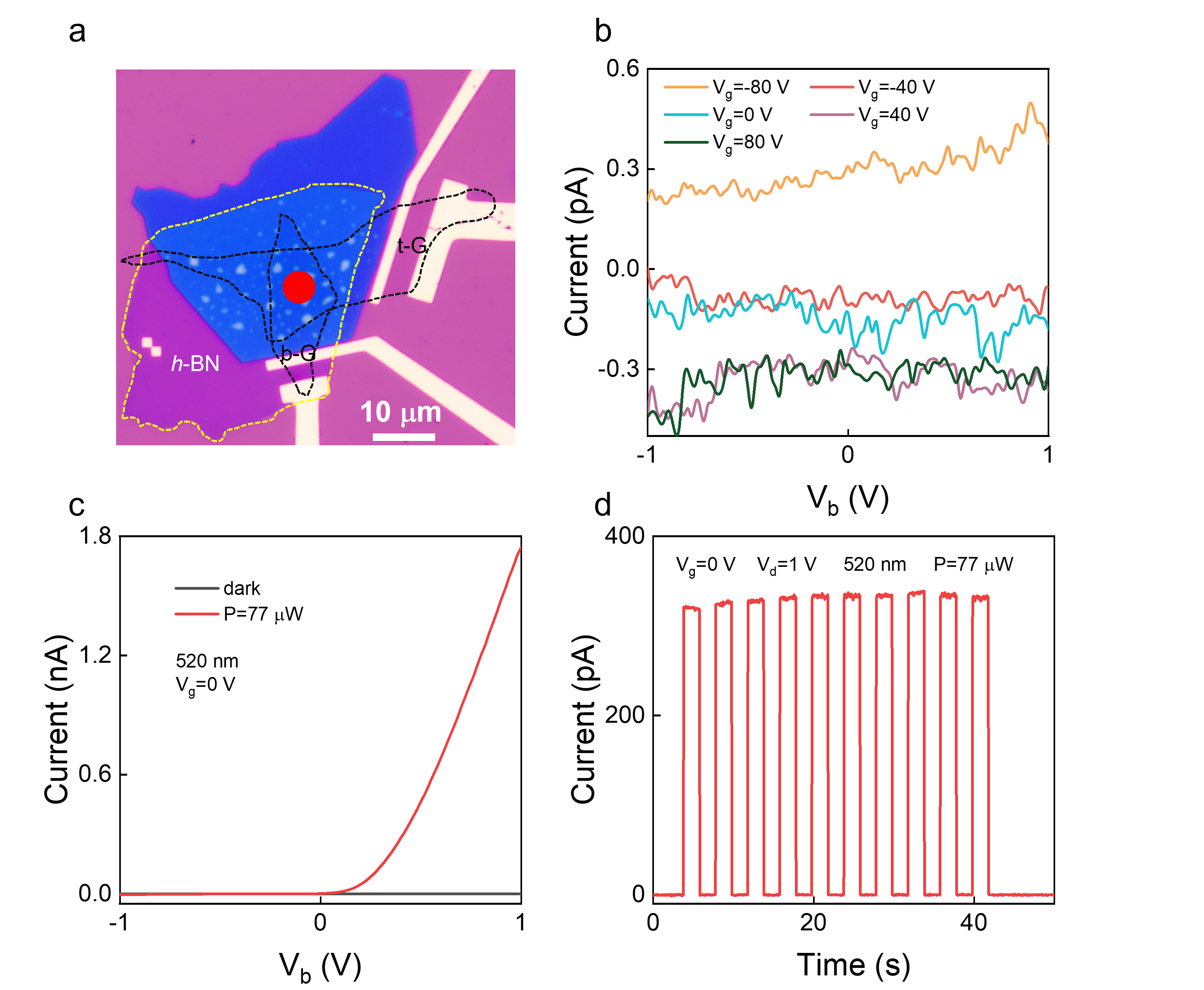


**Figure S17. Characterizations of the few-layer graphene/*h*-BN/few-layer graphene device.** a) Optical image of the device. b) Output characteristic curves of the device with different V_g_, showing the excellent insulating behavior of *h*-BN. c) Output characteristic of the device with V_g_=0 V in the dark and under the irradiation of a 77 µW, 520 nm laser. d) Endurance measurement of the photo current at V_g_=0 V and V_d_=1V.

**
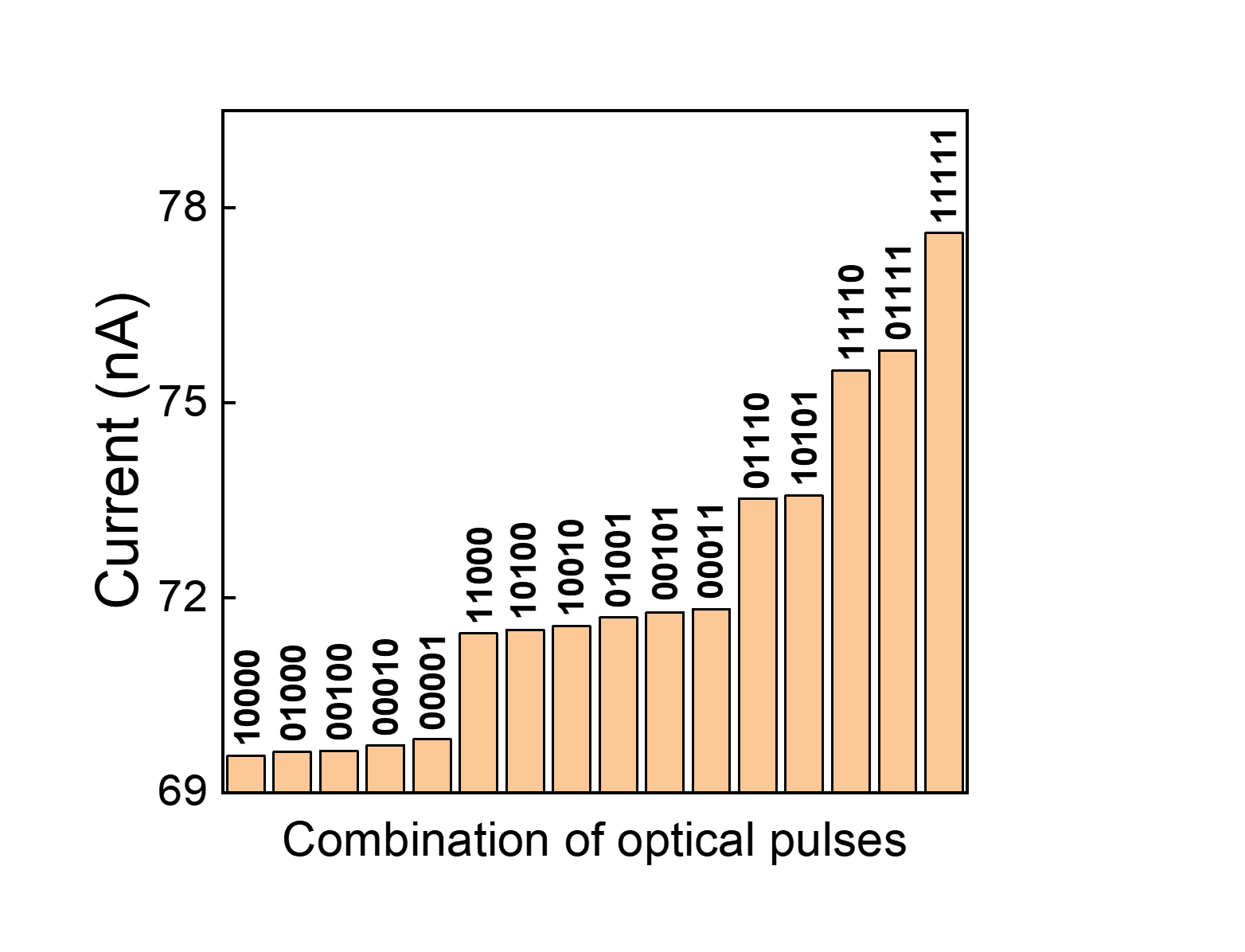
**

**Figure S18. The *I-t* plots correspond to 16 different optical pulse combinations, with 16 distinct current values that can be distinguished.**

**
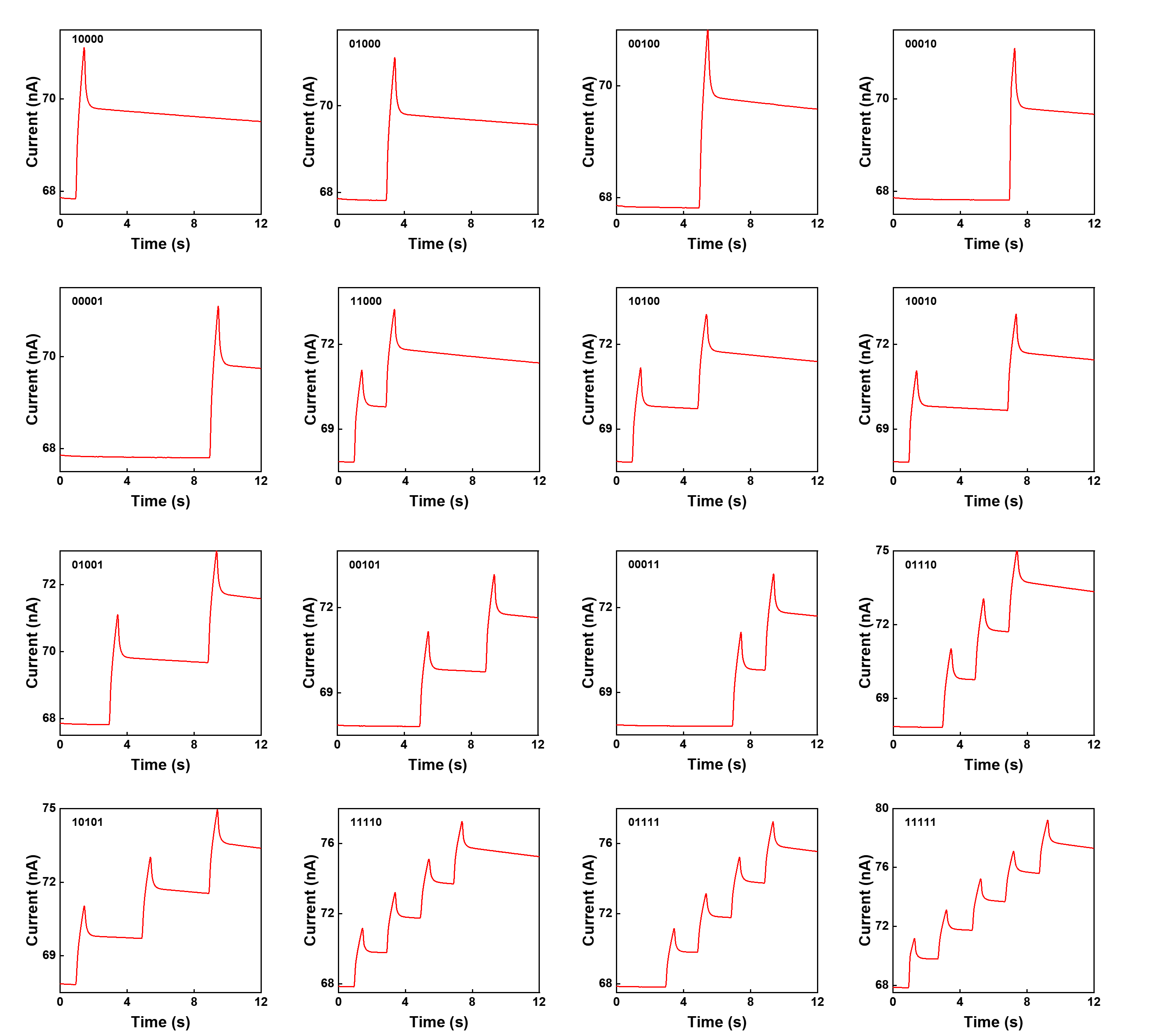
**

**Figure S19. All the *I-t* photo-response characteristics of the inputs used in our experiment to recognize the arrow direction.**

**
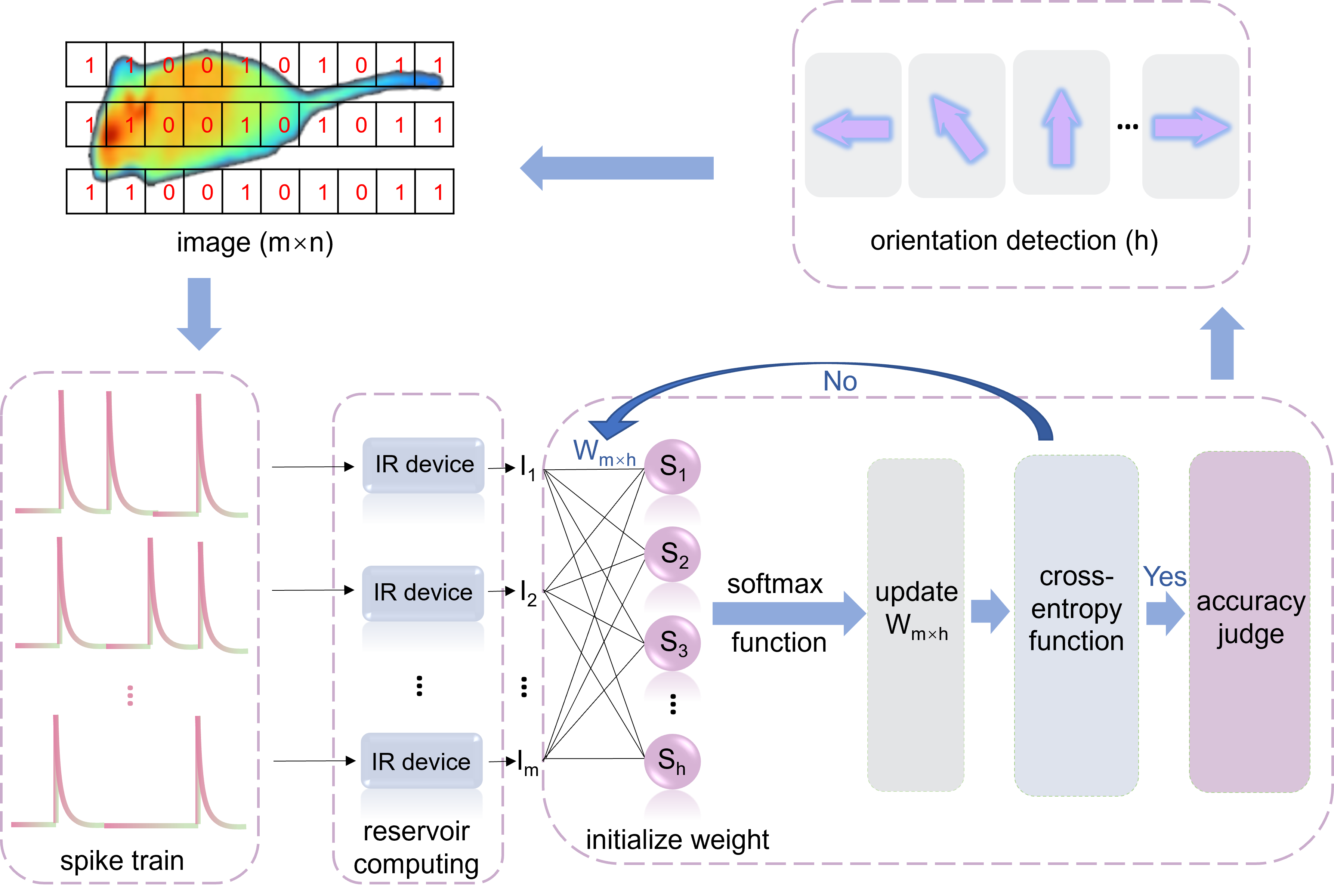
**

**Figure S20. The RC process for orientation classification recognition.**

| Structure | Synaptic functions | Wavelength (nm) | PPF index (%) | Mechanism of optical synapse |
| --- | --- | --- | --- | --- |
| graphene/CuInP_2_S_6_/Au^[1]^ | PPF、EPSC、LTP、LTD、STM、LTM | 365 | 60 | Photoconductivity |
| MXene/violet phosphorus^[2]^ | PPF、EPSC、STP、LTP | 360 | 135 | Photoconductivity |
| a-GaO_x_^[3]^ | PPF、STM | 254 | 150 | Photoconductivity |
| α-In2Se_3_^[4]^ | PSC、PPF、PPD、LTP、LTD | 655 | 40 | Photoconductivity |
| NbS_2_/MoS_2_^[5]^ | PPF、SNDP | 532 | 135 | Photoconductivity due to gate-tunable charge transfer |
| MoS_2_^[6]^ | STP、LTP、PPF | 310 | 120 | Photoconductivity  and the volatile resistive switching |
| α-InSe_3_/GaSe^[7]^ | PPF、LTP、LTD | 808 | 22 | Photoconductivity and optical gating effect |
| PtSe_2−x_^[8]^ | PPF | 405-980 | 123  182 | Coexistence of the photoconductive effect and bolometric  effect |
| MoSSe^[9]^ | STP、LTP、LTD、PPF、SNDP | 450 | 165 | Photosensitivity |
| graphdiyne/graphene/PbS^[10]^ | EPSC、IPSC、PPF | 450-980 | 190  180 | Photogating effect |
| 2D imine polymer^[11]^ | EPSC、PPF、STM、LTM | 400 | 165 | Photogating effect |
| MoS_2_/PTCDA^[12]^ | STP、LTP、IPSC、EPSC、PPD、PPF、SRDP | 532 | 150 | The gate tunable band  alignment |
| MoS_2_/h-BN/Te^[13]^ | EPSC、PPF | 532 | 240 | Charge trapping |
| graphene/α-In_2_Se_3_/graphene^[14]^ | PPF | 637 | 22 | The ferroelectricity affects |
| (PEA)_2_SnI_4_^[15]^ | STP、LTP、PPF | 470 | 130 | Photogenerated carrier trapping/detrapping |
| α-In_2_Se_3_^[16]^ | PPF | 900 | 1000 | Domain wall motion |
| BP^[17]^ | STM、LTM、PPF | 280 | 280 | Oxidation-related defects |
| IGZO/SnO/SnS^[18]^ | EPSC、IPSC、SNDP、SRDP、PPF | 266-658 | 112  1600 | Generation and recombination of carriers |
| graphene/h-BN/MoS_2_^[19]^ | STP、LTP、PPF | 470 | 270 | Photogenerated carrier tunneling |
| Bi_2_O_2_Se/graphene^[20]^ | STM、LTM、LTP、LTD、PPF、EPSC、IPSC、SRDP | 365-635 | 190  120 | Light-induced surface potential change |
| AlGaN/GaN^[21]^ | EPSC、IPSC、PPF | 532 | 200 | Photo-enhanced field-effect mechanism |
| heterobilayer films from perylene and graphene oxide precursors^[22]^ | EPSC、SIDP、SNDP、PPF、STM、LTM | 365-1550 | 214 | Slow release of trapped carriers  in defects |
| This work | **EPSC、STP、LTP、PPF、SRDP、STDP** | **520-2000** | **184** | **Interface coupling effect** |

**Table S1. Key parameters between our synaptic device and other representative 2D optoelectronic synaptic devices.**

| **Thickness of CrOCl (nm)** | **I_dark_ (nA)** | **Synaptic plasticity** | **ΔA_10_/ΔA_1_ (520 nm)** | **ΔA_10_/ΔA_1_ (1064 nm)** | **PPF (%, Δt=2 s,520 nm)** | **PPF (%, Δt=2 s,1064 nm)** |
| --- | --- | --- | --- | --- | --- | --- |
| 2.5 | 900000 | Yes | 6.78 | 8.79 | 154.91 | 165.69 |
| 16 | 80 | Yes | 7.60 | 7.44 | 146.22 | 179.55 |
| 40 | 6.5 | Yes | 3.03 | 7.96 | 138.29 | 179.46 |

**Table S2. Some parameters between our devices with the CrOCl of 2.5 nm, 16 nm, and 40 nm.**

**References**

[1] Y. Liu, Y. Wu, H. Han, Y. Wang, R. Peng, K. Liu, D. Yi, C. W. Nan, J. Ma, CuInP_2_S_6_-Based Electronic/Optoelectronic Synapse for Artificial Visual System Application. *Adv. Funct. Mater.* **2023**, *34*, 2306945.

[2] H. Ma, H. Fang, X. Xie, Y. Liu, H. Tian, Y. Chai, Optoelectronic Synapses Based on MXene/Violet Phosphorus Van Der Waals Heterojunctions for Visual-Olfactory Crossmodal Perception. *Nano-Micro Lett.* **2024**, *16*, 104.

[3] Z. Zhang, X. Zhao, X. Zhang, X. Hou, X. Ma, S. Tang, Y. Zhang, G. Xu, Q. Liu, S. Long, In-Sensor Reservoir Computing System for Latent Fingerprint Recognition with Deep Ultraviolet Photo-Synapses and Memristor Array. *Nat. Commun.* **2022**, *13*, 6590.

[4] K. Liu, T. Zhang, B. Dang, L. Bao, L. Xu, C. Cheng, Z. Yang, R. Huang, Y. Yang, An Optoelectronic Synapse Based on α-In_2_Se_3_ with Controllable Temporal Dynamics for Multimode and Multiscale Reservoir Computing. *Nat. Electron.* **2022**, *5*, 761.

[5] P.-Y. Huang, B.-Y. Jiang, H.-J. Chen, J.-Y. Xu, K. Wang, C.-Y. Zhu, X.-Y. Hu, D. Li, L. Zhen, F.-C. Zhou, J.-K. Qin, C.-Y. Xu, Neuro-Inspired Optical Sensor Array for High-Accuracy Static Image Recognition and Dynamic Trace Extraction. *Nat. Commun.* **2023**, *14*, 6736.

[6] H.-K. He, R. Yang, W. Zhou, H.-M. Huang, J. Xiong, L. Gan, T.-Y. Zhai, X. Guo, Photonic Potentiation and Electric Habituation in Ultrathin Memristive Synapses Based on Monolayer MoS_2_. *Small* **2018**, *14*, 1800079.

[7] F. Guo, M. L. Song, M.-C. Wong, R. Ding, W. F. Io, S.-Y. Pang, W. J. Jie, J. H. Hao, Multifunctional Optoelectronic Synapse Based on Ferroelectric Van Der Waals Heterostructure for Emulating the Entire Human Visual System. *Adv. Funct. Mater.* **2022**, *32*, 2108014.

[8] Y. Lian, J. Han, M. Yang, S. Peng, C. Zhang, C. Han, X. Zhang, X. Liu, H. Zhou, Y. Wang, C. Lan, J. Gou, Y. Jiang, Y. Liao, H. Yu, J. Wang, Tunable Bi-Directional Photoresponse in Hybrid PtSe_2−x_ Thin Films Based on Precisely Controllable Selenization Engineering. Adv. Funct. Mater. 2022, 32, 2205709.

[9] J. Meng, T. Wang, H. Zhu, L. Ji, W. Bao, P. Zhou, L. Chen, Q.-Q. Sun, D. W. Zhang, Integrated In-Sensor Computing Optoelectronic Device for Environment-Adaptable Artificial Retina Perception Application. Nano Lett. 2022, 22, 81.

[10] Y.-X. Hou, Y. Li, Z.-C. Zhang, J.-Q. Li, D.-H. Qi, X.-D. Chen, J.-J. Wang, B.-W. Yao, M.-X. Yu, T.-B. Lu, J. Zhang, Large-Scale and Flexible Optical Synapses for Neuromorphic Computing and Integrated Visible Information Sensing Memory Processing. *ACS Nano* **2021**, *15*, 1497.

[11] J. Zhang, Q. Shi, R. Wang, X. Zhang, L. Li, J. Zhang, L. Tian, L. Xiong, J. Huang, Spectrum-Dependent Photonic Synapses Based on 2D Imine Polymers for Power-Efficient Neuromorphic Computing. *InfoMat* **2021**, *3*, 904.

[12] S. Wang, C. Chen, Z. Yu, Y. He, X. Chen, Q. Wan, Y. Shi, D. W. Zhang, H. Zhou, X. Wang, P. Zhou, A MoS_2_ /PTCDA Hybrid Heterojunction Synapse with Efficient Photoelectric Dual Modulation and Versatility. *Adv. Mater.* **2019**, *31*, 1806227.

[13] J. Zha, Y. Xia, S. Shi, H. Huang, S. Li, C. Qian, H. Wang, P. Yang, Z. Zhang, Y. Meng, W. Wang, Z. Yang, H. Yu, J. C. Ho, Z. Wang, C. Tan, A 2D Heterostructure-Based Multifunctional Floating Gate Memory Device for Multimodal Reservoir Computing. *Adv. Mater.* **2024**, *36*, 2308502.

[14] J. Zeng, G. Feng, G. Wu, J. Liu, Q. Zhao, H. Wang, S. Wu, X. Wang, Y. Chen, S. Han, B. Tian, C. Duan, T. Lin, J. Ge, H. Shen, X. Meng, J. Chu, J. Wang, Multisensory Ferroelectric Semiconductor Synapse for Neuromorphic Computing. *Adv. Funct. Mater.* **2024**, *34*, 2313010.

[15] Y. Sun, L. Qian, D. Xie, Y. Lin, M. Sun, W. Li, L. Ding, T. Ren, T. Palacios, Photoelectric Synaptic Plasticity Realized by 2D Perovskite. *Adv. Funct. Mater.* **2019**, *29*, 1902538.

[16] F. Xue, X. He, W. Liu, D. Periyanagounder, C. Zhang, M. Chen, C.-H. Lin, L. Luo, E. Yengel, V. Tung, T. D. Anthopoulos, L.-J. Li, J.-H. He, X. Zhang, Optoelectronic Ferroelectric Domain-Wall Memories Made From a Single Van Der Waals Ferroelectric. *Adv. Funct. Mater.* **2020**, *30*, 2004206.

[17] T. Ahmed, S. Kuriakose, E. L. H. Mayes, R. Ramanathan, V. Bansal, M. Bhaskaran, S. Sriram, S. Walia, Optically Stimulated Artificial Synapse Based on Layered Black Phosphorus. *Small* **2019**, *15*, 1900966.

[18] T. Zhang, C. Fan, L. Hu, F. Zhuge, X. Pan, Z. Ye, A Reconfigurable All-Optical-Controlled Synaptic Device for Neuromorphic Computing Applications. *ACS Nano* **2024**, *18*, 16236.

[19] Y. Sun, M. Li, Y. Ding, H. Wang, H. Wang, Z. Chen, D. Xie, Programmable Van-Der-Waals Heterostructure-Enabled Optoelectronic Synaptic Floating-Gate Transistors with Ultra-Low Energy Consumption. *InfoMat* **2022**, *4*, e12317.

[20] C.-M. Yang, T.-C. Chen, D. Verma, L.-J. Li, B. Liu, W.-H. Chang, C.-S. Lai, Bidirectional All-Optical Synapses Based on a 2D Bi_2_O_2_Se/Graphene Hybrid Structure for Multifunctional Optoelectronics. *Adv. Funct. Mater.* **2020**, *30*, 2001598.

[21] X. Hong, Y. Huang, Q. Tian, S. Zhang, C. Liu, L. Wang, K. Zhang, J. Sun, L. Liao, X. Zou, Two-Dimensional Perovskite-Gated AlGaN/GaN High-Electron-Mobility-Transistor for Neuromorphic Vision Sensor. *Adv. Sci.* **2022**, *9*, 2202019.

[22] H.-S. Zhang, X.-M. Dong, Z.-C. Zhang, Z.-P. Zhang, C.-Y. Ban, Z. Zhou, C. Song, S.-Q. Yan, Q. Xin, J.-Q. Liu, Y.-X. Li, W. Huang, Co-Assembled Perylene/Graphene Oxide Photosensitive Heterobilayer for Efficient Neuromorphics. *Nat. Commun.* **2022**, *13*, 4996.
